# Supplementary material for: Dual asparagine-depriving nanoparticles against solid tumors
Source: Nat Commun. 2025 Jul 1;16:5675. doi: 10.1038/s41467-025-60798-y (PMC12215482; doi:10.1038/s41467-025-60798-y)
Supplement: Supplementary file 1 — Supplementary Information [file 41467_2025_60798_MOESM1_ESM.pdf]

Supporting Information

Dual Asparagine-Depriving Nanoparticles Against Solid Tumors

Yubo Shen,[1] Huifang Wang,[1] Daoxia Guo,[1] Jiantao Liu,[2] Jinli Sun,[1] Nan Chen,[2,*]

Haiyun Song,[1,*] Xiaoyuan Ji[1,*]

[1]School of Public Health, Shanghai Jiao Tong University School of Medicine, Shanghai 200025, China

[2]College of Chemistry and Materials Science, The Education Ministry Key Lab of Resource Chemistry, Joint International Research Laboratory of Resource Chemistry of Ministry of Education, Shanghai Key Laboratory of Rare Earth Functional Materials, and Shanghai Frontiers Science Center of Biomimetic Catalysis, Shanghai Normal University, Shanghai 200234, China

*Correspondence: Nan Chen, nchen@shnu.edu.cn

Haiyun Song, songhaiyun@shsmu.edu.cn

Xiaoyuan Ji, xyji@shsmu.edu.cn

This supplemental file includes:

Supplementary Figures 1-41

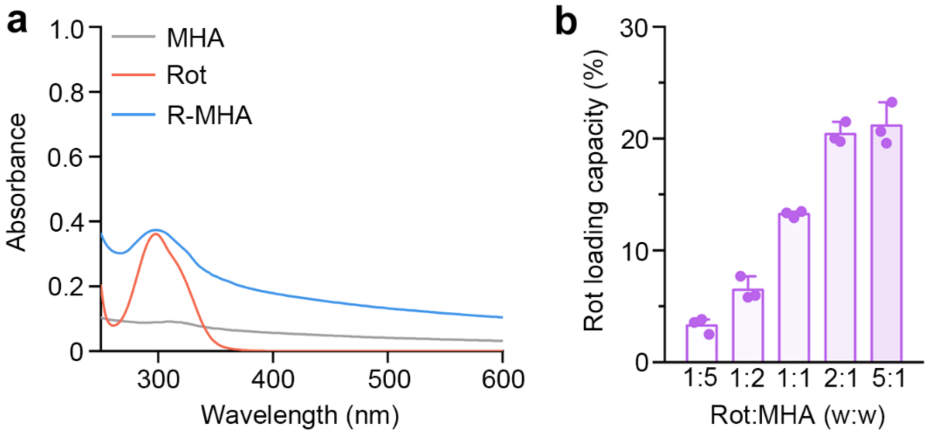

Supplementary Figure 1. Characterization of R-MHAs. (a) UV-Vis absorption spectra of free Rot, unloaded MHAs and R-MHAs. (b) Drug loading capacity of MHAs at various Rot to MHA mass ratios. Data are represented as mean ± SD (n = 3 independent samples). Rot rotenone. Source data are provided as a Source Data file.

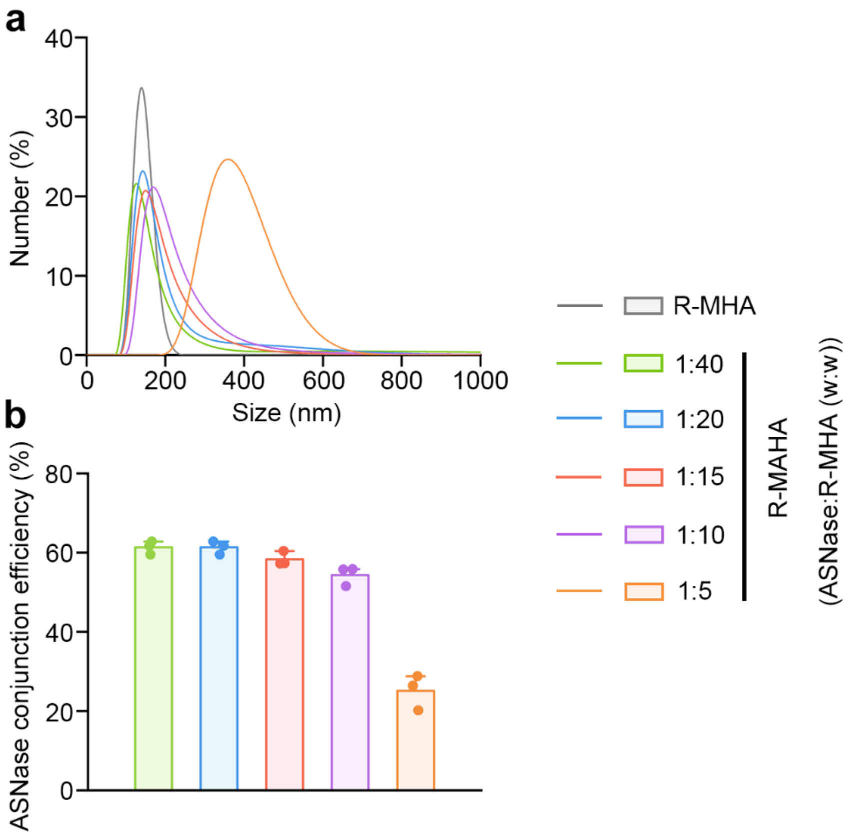

Supplementary Figure 2. Formation of cross-linked ASNase shell on R-MHAs. (a) Hydrodynamic diameters of R-MHAs and R-MAHAs prepared at various ASNase to R-MHA mass ratios. (b) Conjugation efficiency of ASNase in R-MAHAs at various ASNase to R-MHA mass ratios. Data are represented as mean ± SD (n = 3 independent samples). ASNase L-asparaginase. Source data are provided as a Source Data file.

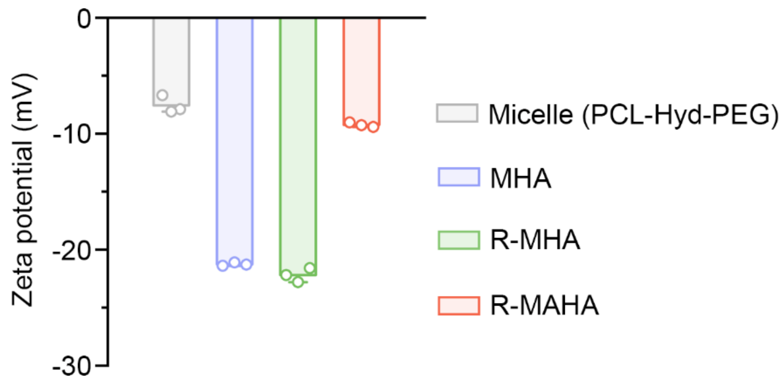

Supplementary Figure 3. Zeta potentials of indicated NPs. Data are represented as mean ± SD (n = 3 independent samples). NPs nanoparticles. Source data are provided as a Source Data file.

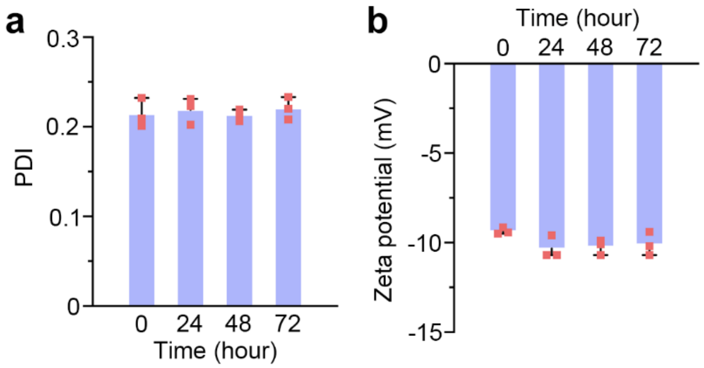

Supplementary Figure 4. Polymer dispersity index (PDI) (a) and zeta potentials (b) of R-MAHAs at different time points in cell culture medium. Data are represented as mean ± SD (n = 3 independent samples). Source data are provided as a Source Data file.

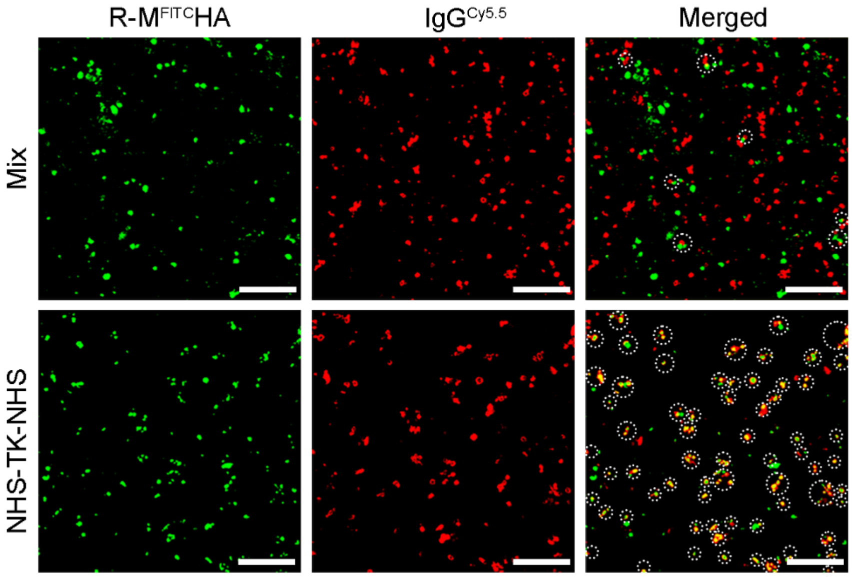

Supplementary Figure 5. Representative confocal fluorescence imaging showing the colocalization of IgG$^{Cy5.5}$ with R-M^{FITC}HAs in the presence or absence of NHS-TK-NHS. Scale bars: 10 μm. NHS-TK-NHS *N*-hydroxy succinimide-thioketal-*N*-hydroxy succinimide, FITC fluorescein isothiocyanate, Cy5.5 cyanine 5.5.

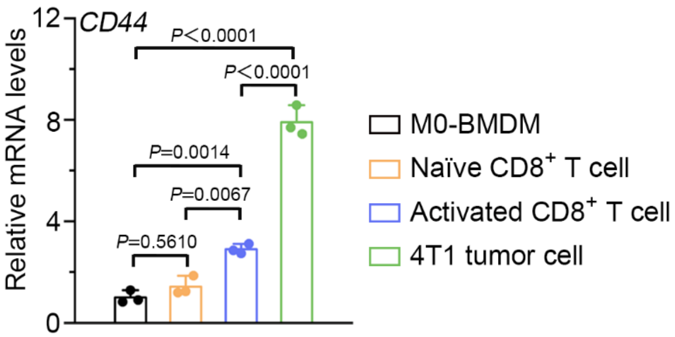

Supplementary Figure 6. The levels of *CD44* expression in different types of cells. Data are represented as mean ± SD (n = 3 independent samples). *P*-values were calculated using a one-way ANOVA followed by Tukey's post-hoc test. BMDM bone marrow-derived macrophages. Source data are provided as a Source Data file.

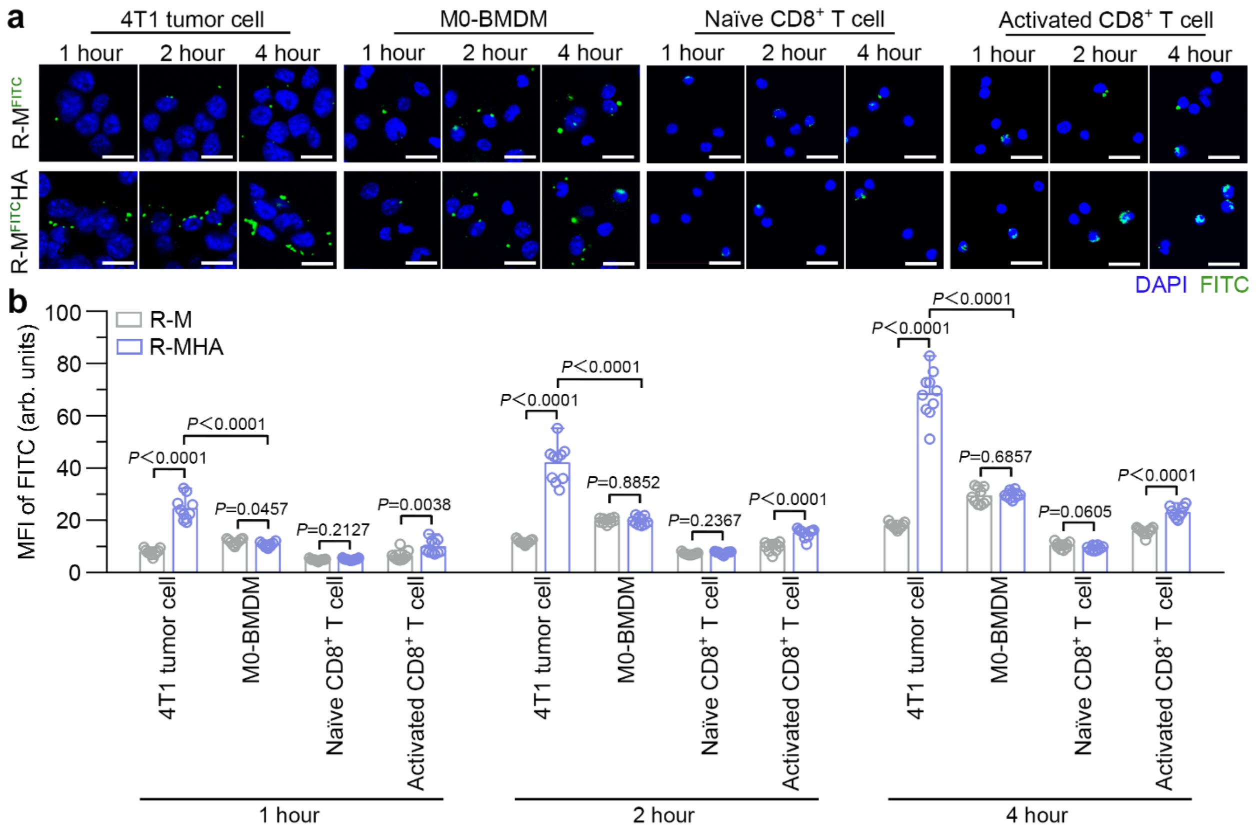

Supplementary Figure 7. Cellular uptake of FITC-labeled NPs. (a) Confocal fluorescence imaging of intracellular levels of R-micelles^{FITC} (R-M^{FITC}) or R-M^{FITC}HAs in 4T1 cells, M0-BMDMs, naïve CD8^{+} T cells, and activated CD8^{+} T cells at various time points. Scale bars: 20 μm. (b) Quantifications of intracellular levels of FITC-labeled NPs. Data are represented as mean ± SD (n = 10 independent samples). *P*-values were calculated using an unpaired two-tailed Student's *t*-test. BMDM bone marrow-derived macrophages, FITC fluorescein isothiocyanate. Source data are provided as a Source Data file.

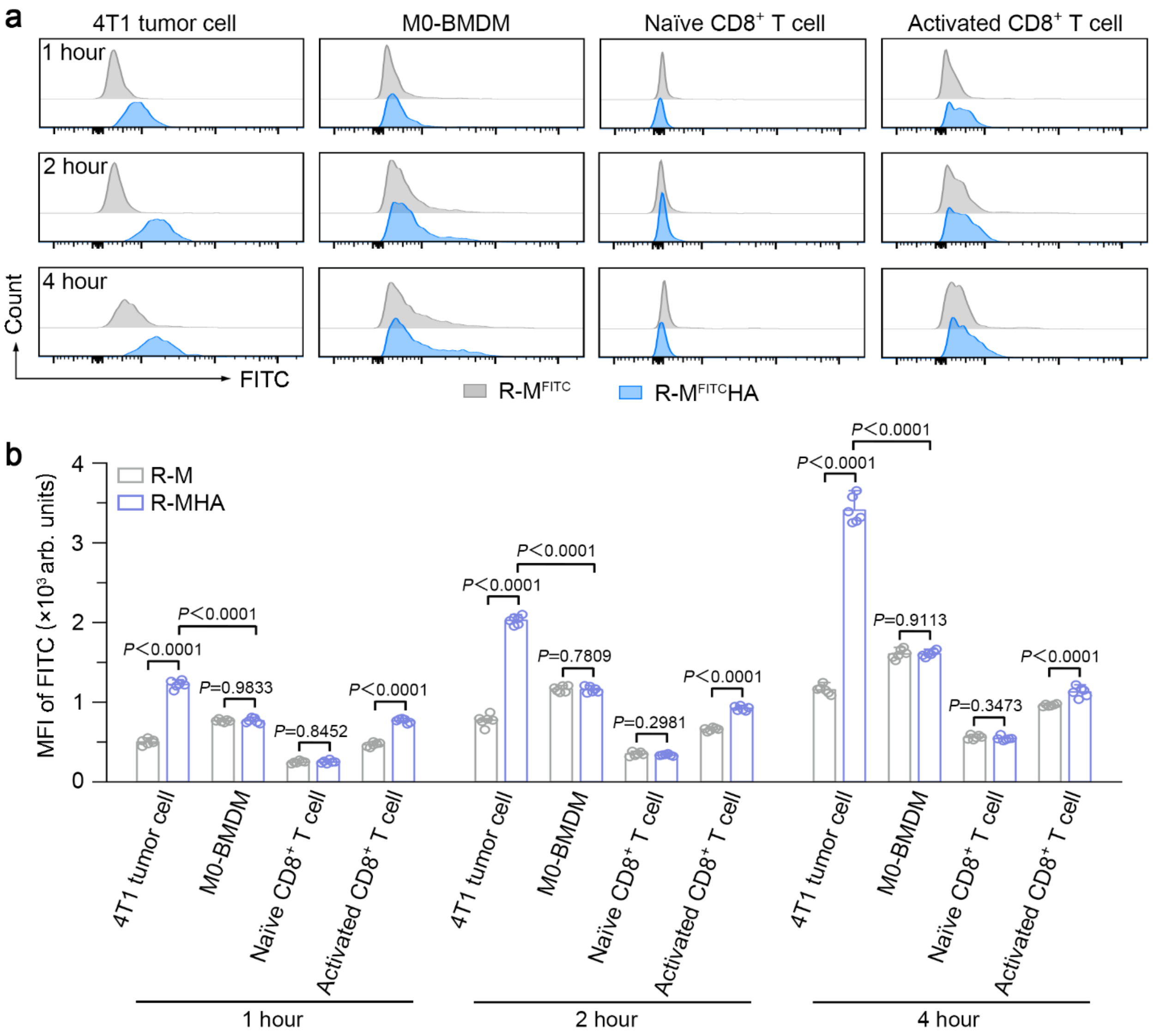

Supplementary Figure 8. Flow cytometric analysis of cellular uptake of FITC-labeled NPs. (a) Representative flow cytometric plots showing the uptake of R-M^{FITC} or R-M^{FITC}HAs by 4T1 cells, M0-BMDMs, naïve CD8^{+} T cells, and activated CD8^{+} T cells at various time points. (b) Quantifications of intracellular levels of FITC-labeled NPs. Data are represented as mean ± SD (n = 6 independent samples). *P*-values were calculated using an unpaired two-tailed Student's *t*-test. BMDM bone marrow-derived macrophages, FITC fluorescein isothiocyanate. Source data are provided as a Source Data file.

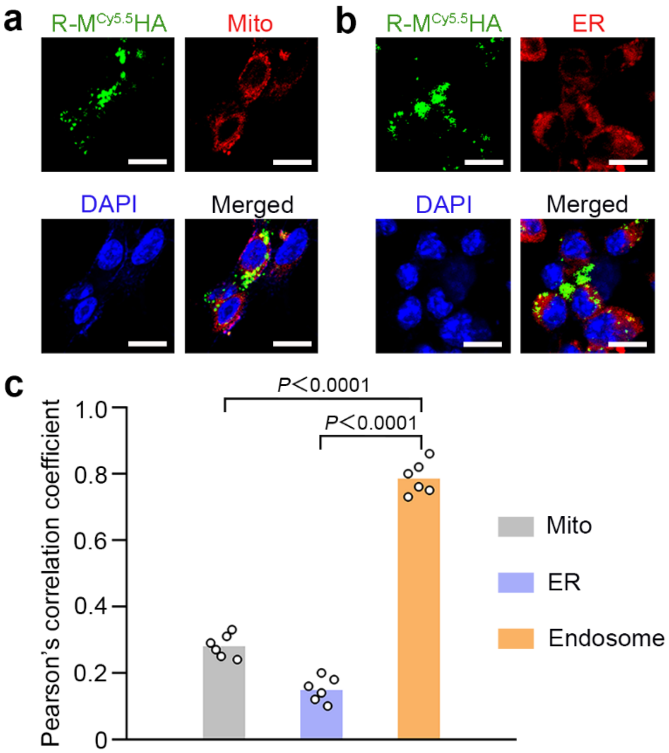

Supplementary Figure 9. Intracellular localization of R-M$^{Cy5.5}$HAs. (a, b) Confocal fluorescence imaging of intracellular R-M$^{Cy5.5}$HAs in 4T1 cells. Mito-Tracker or ER-Tracker was used to label the mitochondria (Mito) or endoplasmic reticulum (ER). Scale bars: 10 μm. (c) Values of Pearson's correlation coefficient between R-MHAs and Mito, ER, or endosome. Data are represented as mean ± SD (n = 6 independent samples). *P*-values were calculated using a one-way ANOVA followed by Tukey's post-hoc test. Cy5.5 cyanine 5.5. Source data are provided as a Source Data file.

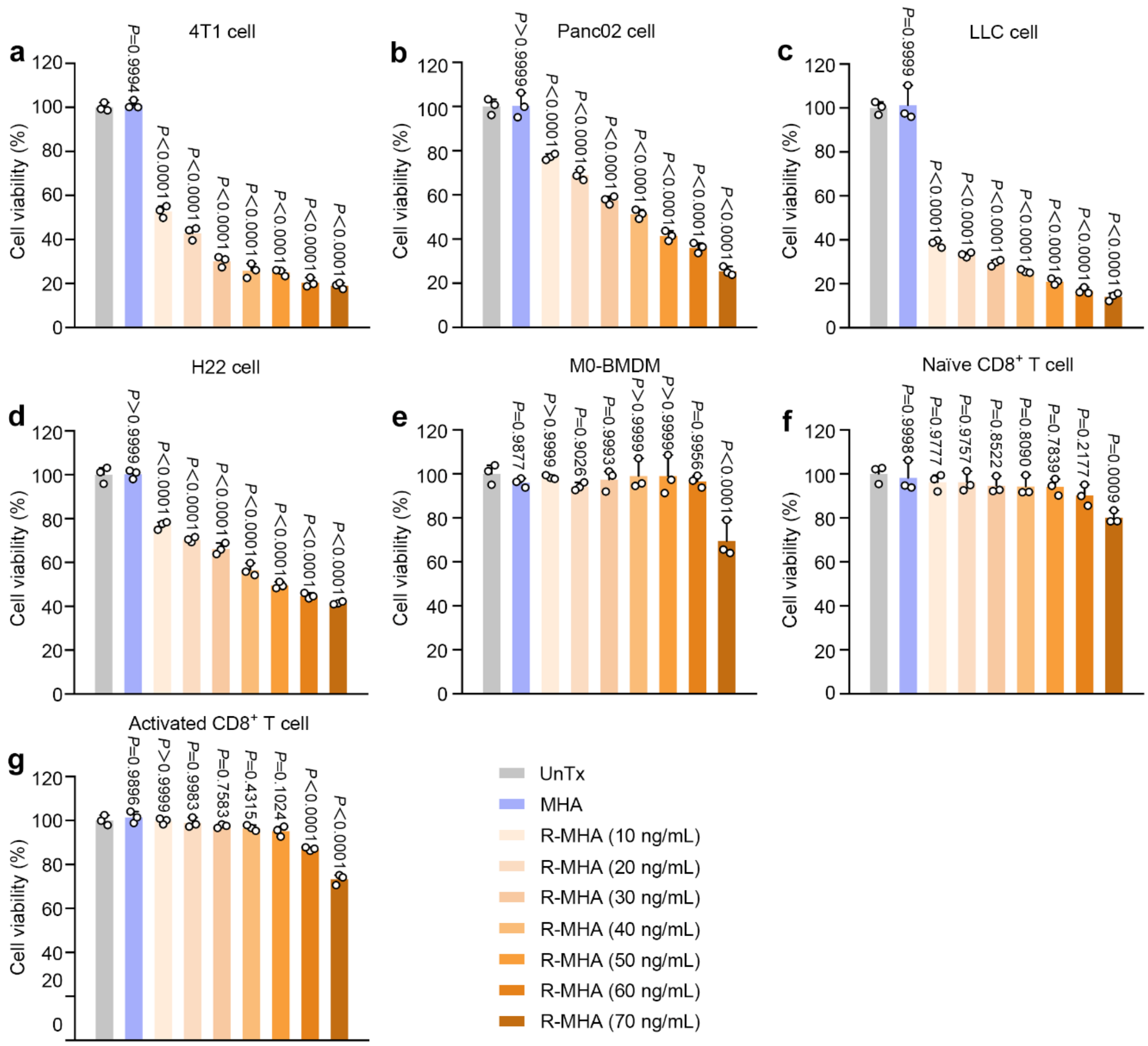

Supplementary Figure 10. Cytotoxicity of R-MHAs on tumor cells and immune cells. Viabilities of 4T1 cells (a), Panc02 cells (b), LLC cells (c), H22 cells (d), M0-BMDMs (e), naïve $CD8^+$ T cells (f), and activated $CD8^+$ T cells (g) after 48 hours of indicated treatment. Data are represented as mean ± SD (n = 3 independent samples). *P*-values were calculated using a one-way ANOVA followed by Tukey's post-hoc test. BMDM bone marrow-derived macrophages. Source data are provided as a Source Data file.

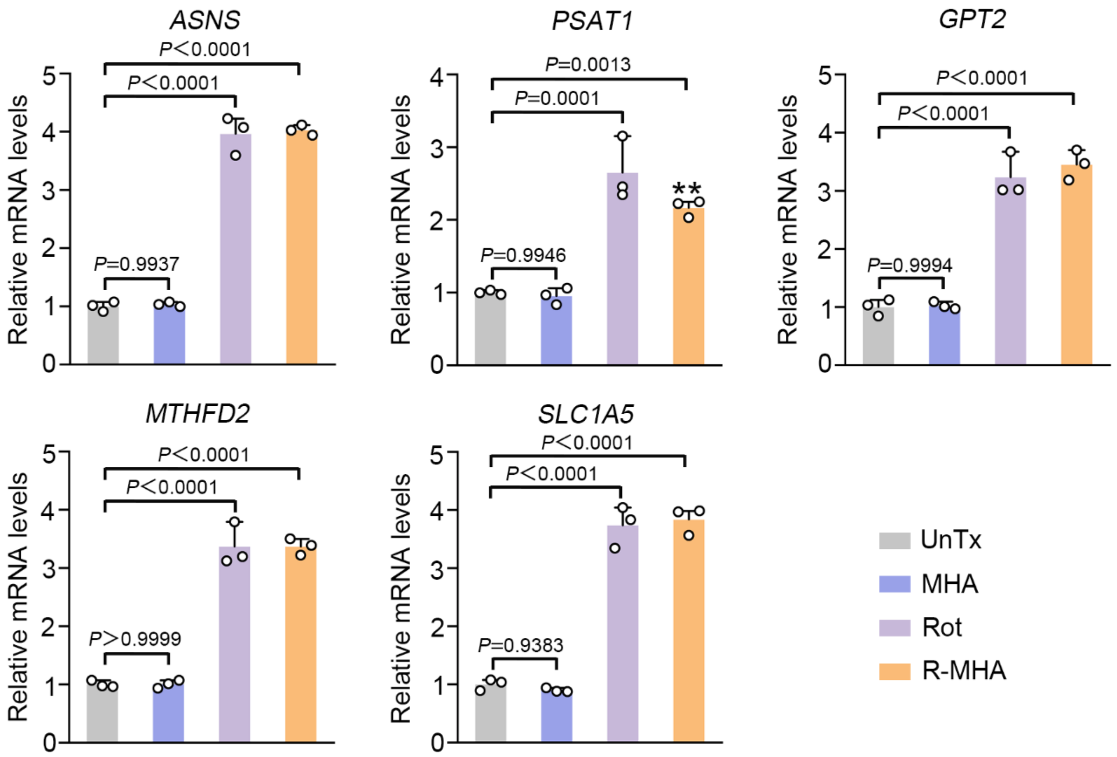

Supplementary Figure 11. mRNA levels of *ASNS*, *PSAT1*, *GPT2*, *MTHFD2*, and *SLC1A5* in 4T1 cells following the indicated treatments. Data are represented as mean ± SD (n = 3 independent samples). *P*-values were calculated using a one-way ANOVA followed by Tukey's post-hoc test. Rot rotenone. Source data are provided as a Source Data file.

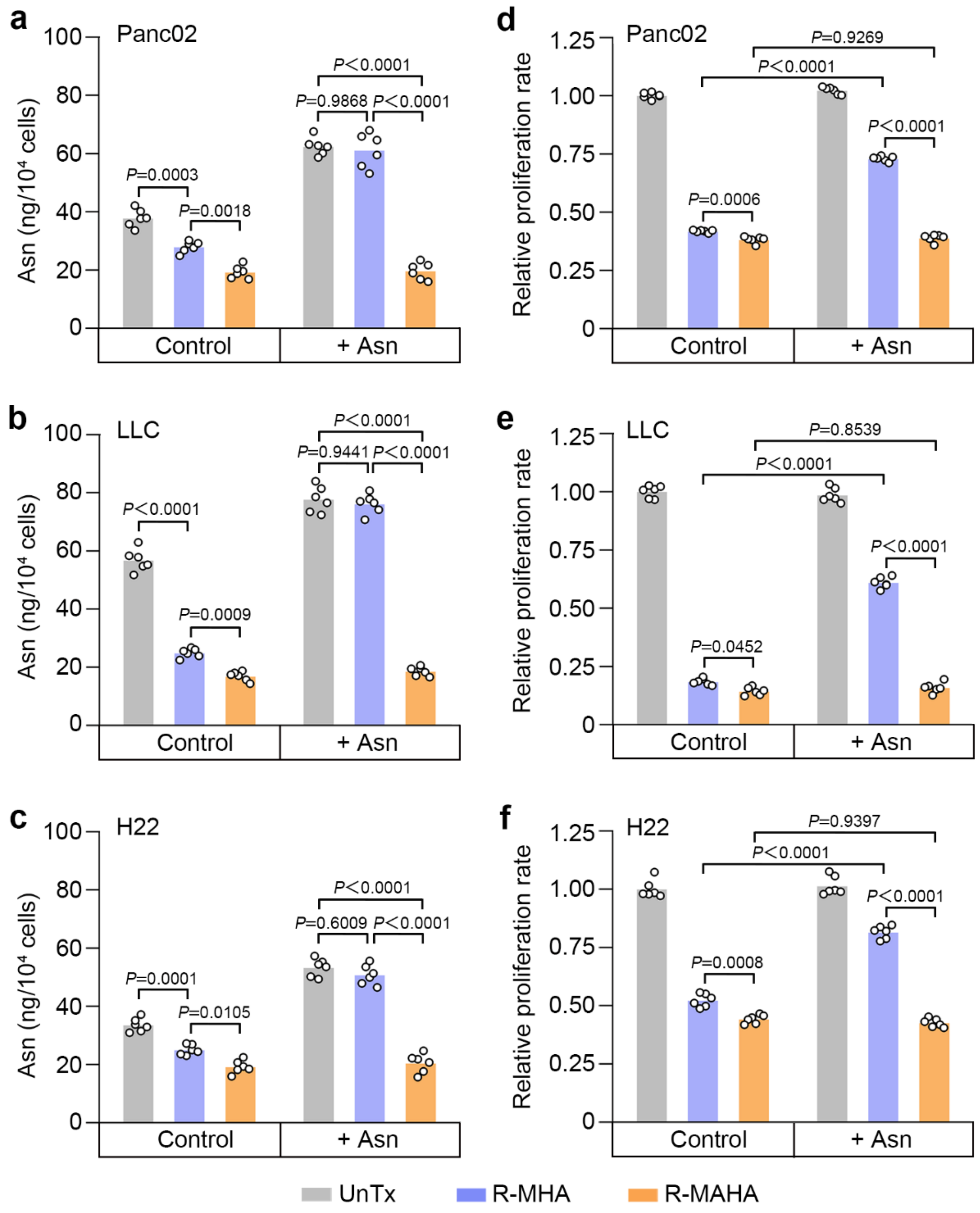

Supplementary Figure 12. Intracellular levels of Asn (a-c) and relative proliferation rates (d-f) of Panc02 cells, LLC cells, and H22 cells after the indicated treatment, with or without supplementing Asn (0.1 mM) in the cell culture medium. R-MHAs (Rot: 50 ng/mL) and R-MAHAs (Rot: 50 ng/mL; ASNase: 0.6 μg/mL) were used. Data are represented as mean ± SD (n = 6 independent samples). *P*-values were calculated using a one-way ANOVA followed by Tukey's post-hoc test. Asn asparagine, ASNase L-asparaginase. Source data are provided as a Source Data file.

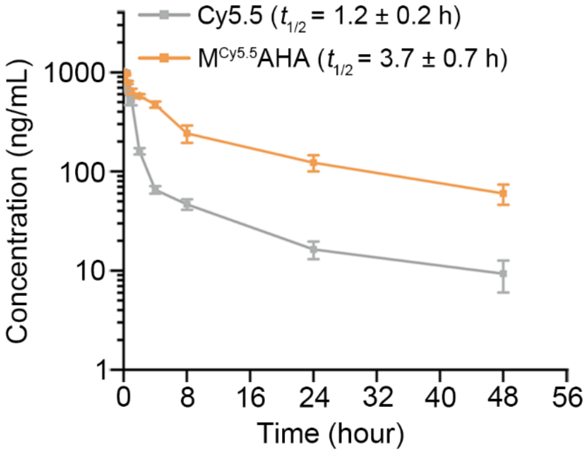

Supplementary Figure 13. In vivo pharmacokinetic curves of free Cy5.5 and $M^{Cy5.5}$AHAs. Free Cy5.5 or $M^{Cy5.5}$AHAs were administered via tail vein injection at a dose of 1 mg/kg Cy5.5. Data are represented as mean ± SD (n = 3 independent samples). Cy5.5 cyanine 5.5. Source data are provided as a Source Data file.

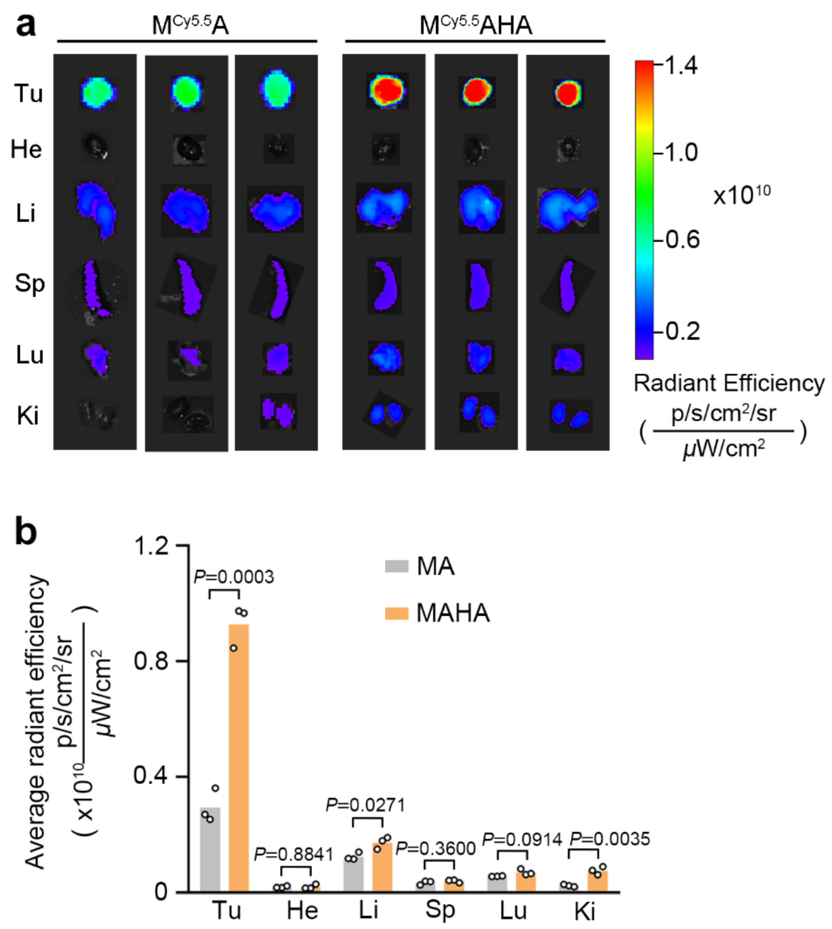

Supplementary Figure 14. Tumor targeting capacity of HA-decorated NPs. (a) Ex vivo fluorescence imaging of tumors and major organs 24 hours after intravenous injection of $M^{Cy5.5}$As or $M^{Cy5.5}$AHAs at a dose of 1 mg/kg Cy5.5. (b) Quantification of fluorescence intensities in tumors and major organs. Data are represented as mean ± SD (n = 3 independent samples). *P*-values were calculated using an unpaired two-tailed Student's *t*-test. Tu tumor, He heart, Li liver, Sp spleen, Lu lung, Ki kidney. Source data are provided as a Source Data file.

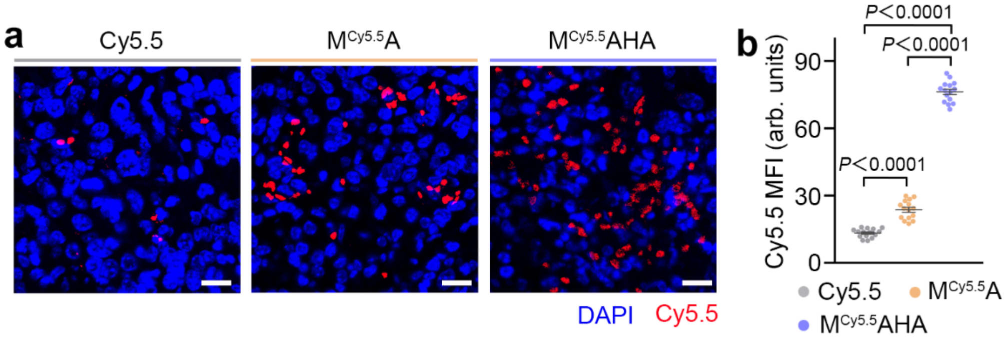

Supplementary Figure 15. Intratumoral accumulation of HA-decorated NPs. (a) Confocal fluorescence imaging of Cy5.5, $M^{Cy5.5}$As or $M^{Cy5.5}$AHAs treated tumor sections. Scale bars: 15 μm. (b) Quantification of fluorescence intensities in tumor sections. Data are represented as mean ± SD (n = 15 independent samples). *P*-values were calculated using a one-way ANOVA followed by Tukey's post-hoc test. Source data are provided as a Source Data file.

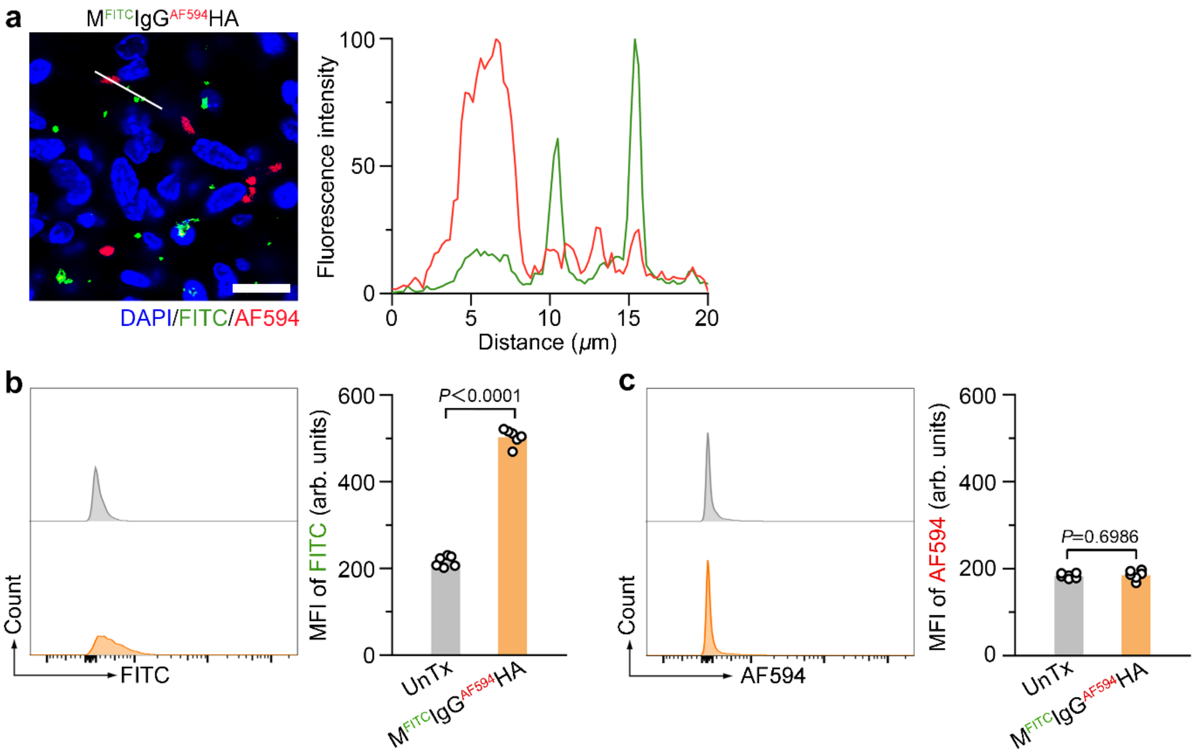

Supplementary Figure 16. Intratumor detachment of core-shell structured NPs. (a) Left: Confocal fluorescence imaging of tumor sections after administration of M^{FITC}IgG^{AF594}HAs. Scale bar: 15 μm. Right: The colocalization of IgG^{AF594} with the M^{FITC}HAs. (b, c) Flow cytometric analysis of M^{FITC}HA and IgG^{AF594} signals in tumor cells (gated on CD45$^-$EpCAM$^+$ population). Data are represented as mean ± SD (n = 6 independent samples). *P*-values were calculated using an unpaired two-tailed Student's *t*-test. FITC fluorescein isothiocyanate, AF594 AlexaFluor 594. Source data are provided as a Source Data file.

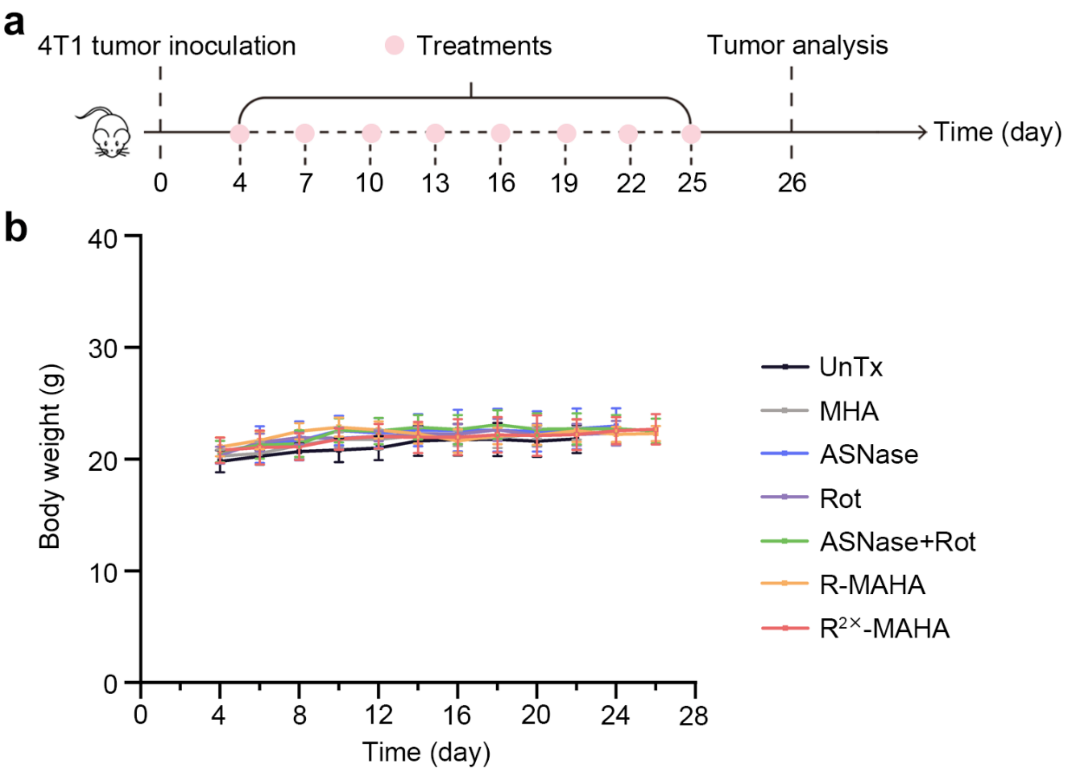

Supplementary Figure 17. (a) Schematic illustration of experimental schedule. (b) Body weight of mice after indicated treatment. Data are represented as mean ± SD (n = 6 mice). ASNase L-asparaginase, Rot rotenone. Source data are provided as a Source Data file.

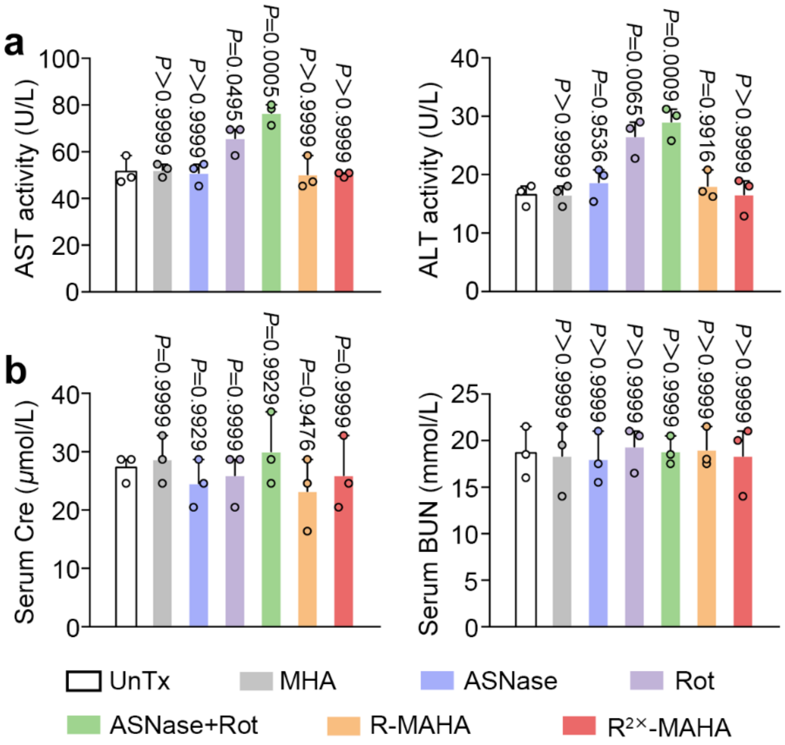

Supplementary Figure 18. Effects of R-MAHAs on serum levels of liver and kidney function parameters. (a) Serum ALT and AST activities in tumor-bearing mice after indicated treatment. (b) Serum levels of Cre and BUN in tumor-bearing mice after indicated treatment. Data are represented as mean ± SD (n = 3 independent samples). *P*-values were calculated using a one-way ANOVA followed by Tukey's post-hoc test. ASNase L-asparaginase, Rot rotenone, ALT alanine transaminase, AST aspartate transaminase, Cre creatinine, BUN blood urea nitrogen. Source data are provided as a Source Data file.

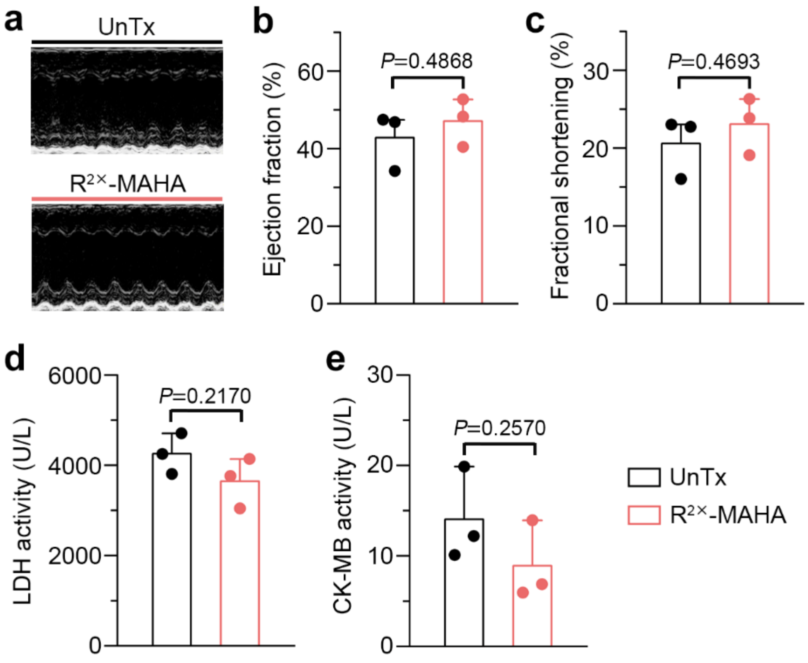

Supplementary Figure 19. Cardiac functional tests. (a) Representative M-mode echocardiography images of tumor-bearing mice after indicated treatment. (b, c) Echocardiographic analysis of ejection fraction and fractional shortening. Data are represented as mean ± SD (n = 3 mice) (d, e) Serum LDH and CK-MB activities in tumor-bearing mice after indicated treatment. Data are represented as mean ± SD (n = 3 independent samples). *P*-values were calculated using an unpaired two-tailed Student's *t*-test. LDH lactate dehydrogenase, CK-MB creatine kinase-MB. Source data are provided as a Source Data file.

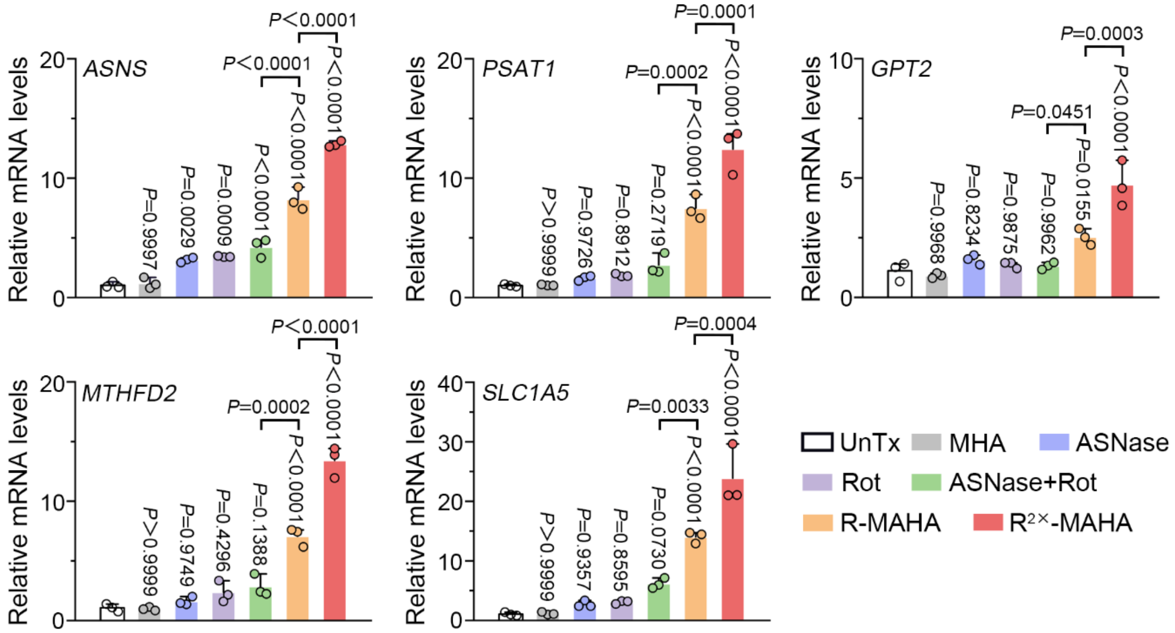

Supplementary Figure 20. Expression of ATF4-driven metabolic genes in tumor cells isolated from 4T1 tumors. Data are represented as mean ± SD (n = 3 independent samples). *P*-values were calculated using a one-way ANOVA followed by Tukey's post-hoc test. ASNase L-asparaginase, Rot rotenone. Source data are provided as a Source Data file.

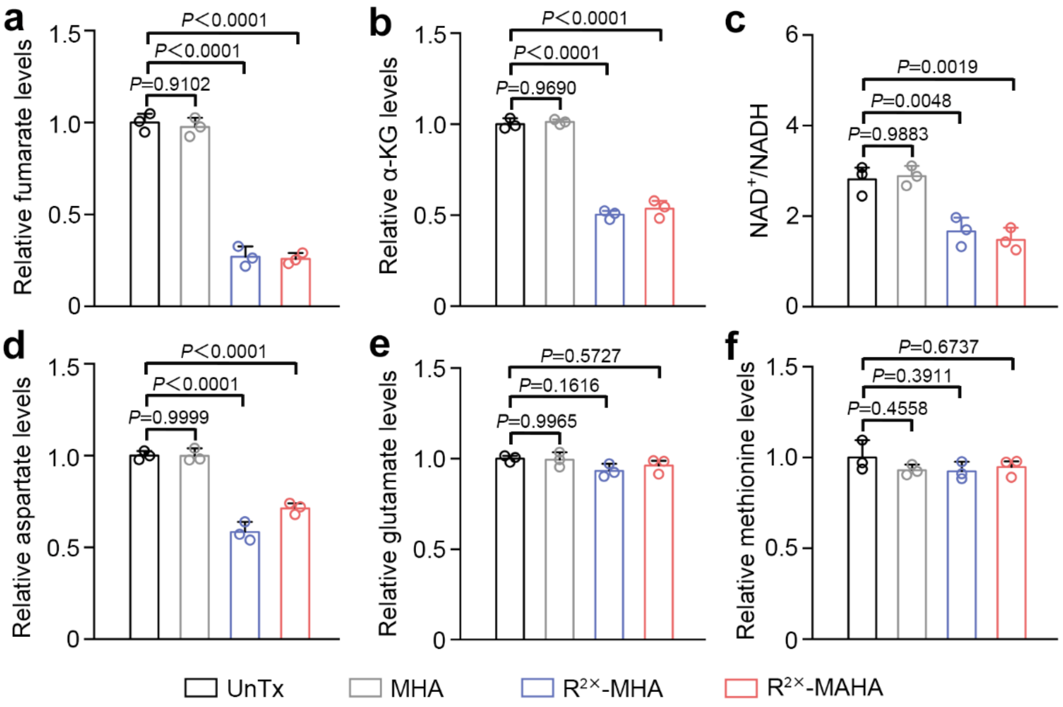

Supplementary Figure 21. Effects of Rot-loaded NPs on TCA cycle metabolites, aspartate, glutamate, and methionine. Levels of fumarate (a), α-KG (b), NAD^+/NADH (c), aspartate (d), glutamate (e), and methionine (f) in 4T1 tumors after indicated treatment. Data are represented as mean ± SD (n = 3 independent samples). *P*-values were calculated using a one-way ANOVA followed by Tukey's post-hoc test. α-KG α-ketoglutaric acid, NAD^+ nicotinamide adenine dinucleotide. Source data are provided as a Source Data file.

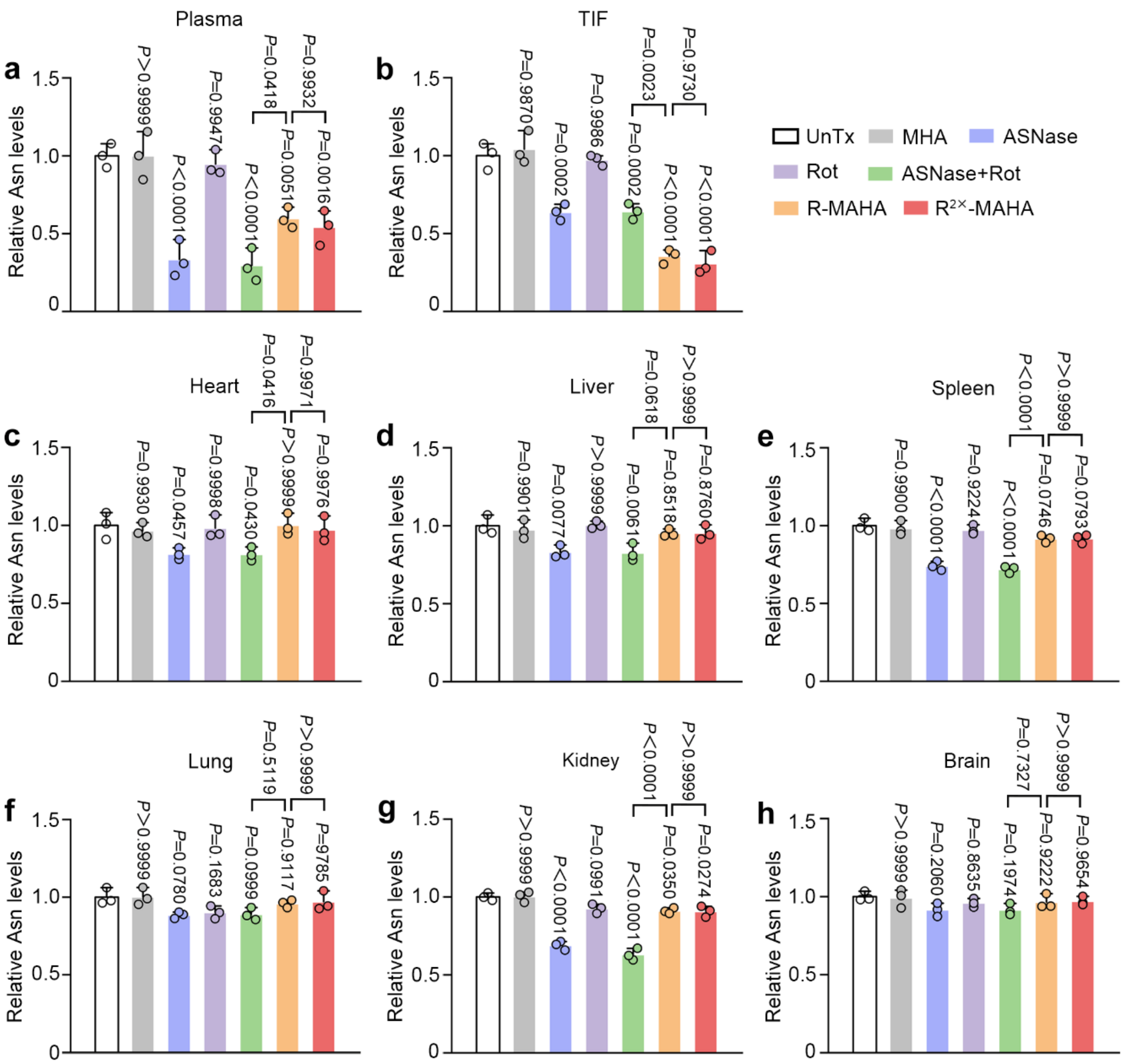

Supplementary Figure 22. The Asn levels in plasma (a), TIF (b), and major organs (c-h) after indicated treatment. Data are represented as mean ± SD (n = 3 independent samples). *P*-values were calculated using a one-way ANOVA followed by Tukey's post-hoc test. ASNase L-asparaginase, Rot rotenone, TIF tumor interstitial fluid. Source data are provided as a Source Data file.

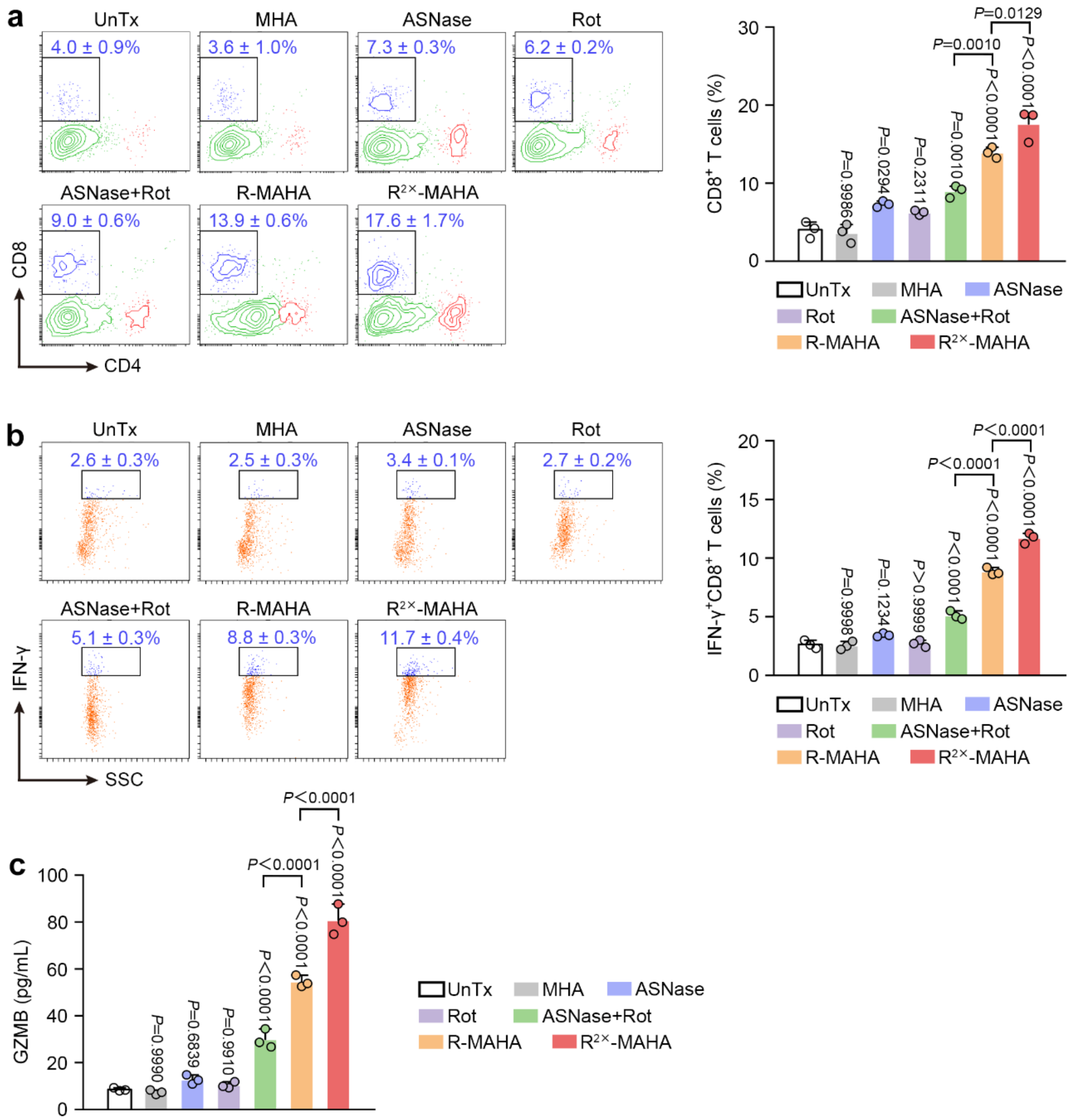

Supplementary Figure 23. Effects of dual Asn-depriving NPs on tumor-infiltrating CD8$^+$ T cells. (a) Flow cytometric analysis (left) and the ratios (right) of CD8$^+$ T cells (gated on CD45$^+$ population) after indicated treatment. (b) Flow cytometric analysis (left) and the ratios (right) of IFN-γ$^+$CD8$^+$ T cells (gated on CD45$^+$CD8$^+$ population) after indicated treatment. (c) Serum levels of GZMB in tumor-bearing mice after indicated treatment. Data are represented as mean ± SD (n = 3 independent samples). *P*-values were calculated using a one-way ANOVA followed by Tukey's post-hoc test. ASNase L-asparaginase, Rot rotenone, IFN-γ interferon-γ, GZMB granzyme B. Source data are provided as a Source Data file.

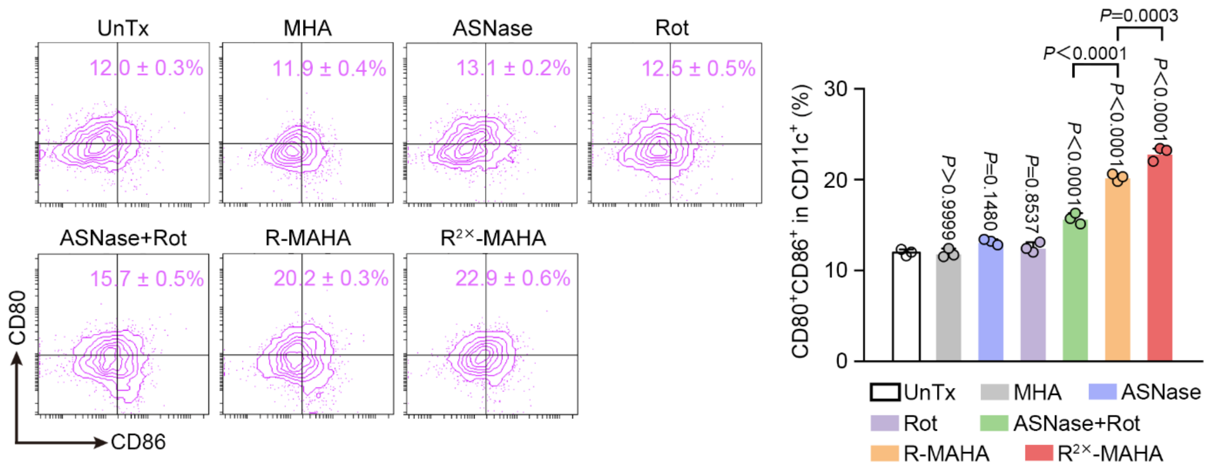

Supplementary Figure 24. Effects of dual Asn-depriving NPs on DC maturation. Flow cytometric analysis (left) and the ratios (right) of $CD80^+CD86^+$ DCs (gated on $CD45^+CD11c^+$ population) after indicated treatment. Data are represented as mean ± SD (n = 3 independent samples). *P*-values were calculated using a one-way ANOVA followed by Tukey's post-hoc test. ASNase L-asparaginase, Rot rotenone. Source data are provided as a Source Data file.

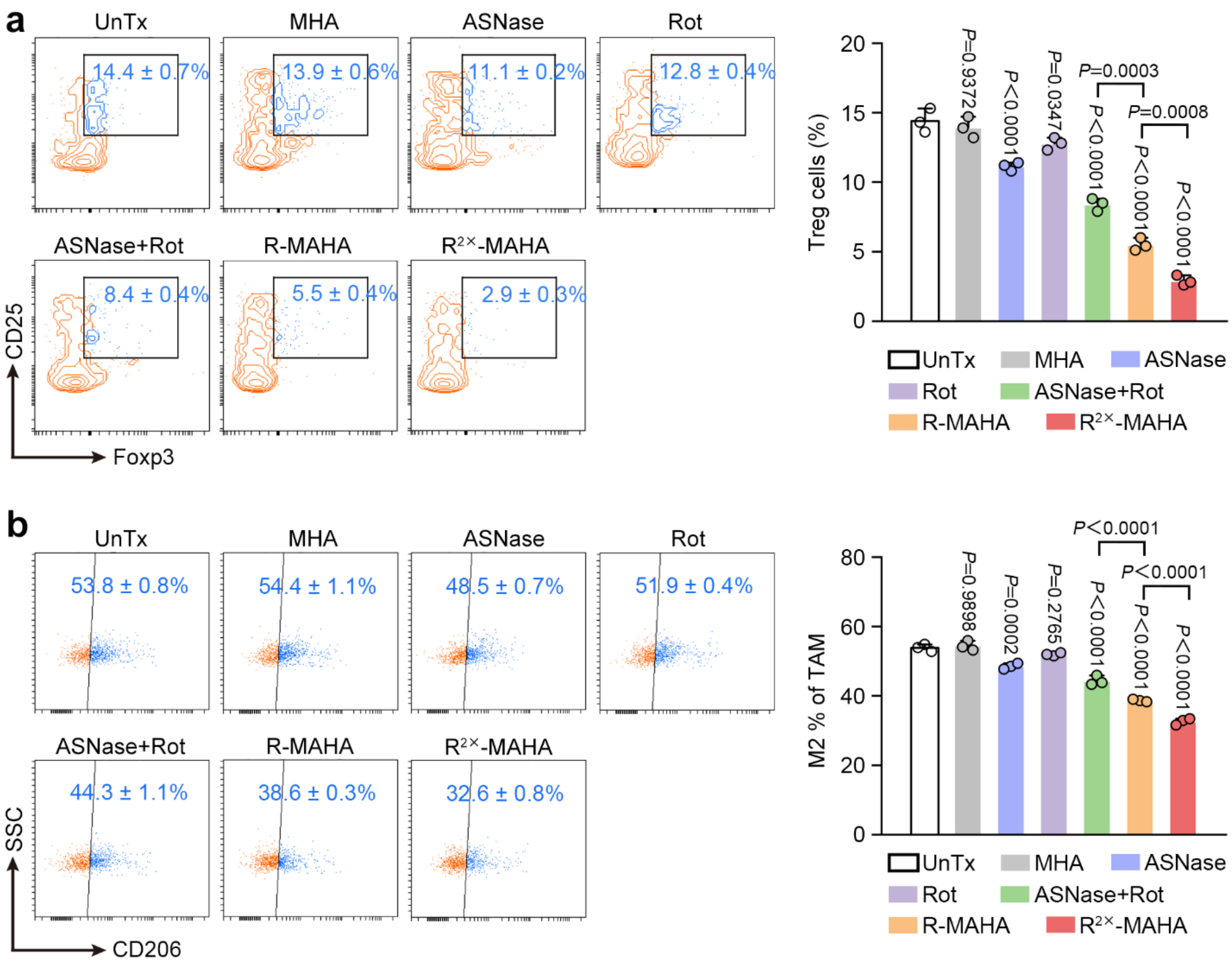

Supplementary Figure 25. Effects of dual Asn-depriving NPs on tumor-infiltrating Treg cells and M2-TAMs. (a) Flow cytometric analysis (left) and the ratios (right) of Treg cells (gated on $CD45^+CD4^+$ population) after indicated treatment. (b) Flow cytometric analysis (left) and the ratios (right) of M2-TAMs (gated on $CD45^+CD11b^+F4/80^+$ population) after indicated treatment. Data are represented as mean ± SD (n = 3 independent samples). *P*-values were calculated using a one-way ANOVA followed by Tukey's post-hoc test. ASNase L-asparaginase, Rot rotenone, Treg regulatory T, TAM tumor-associated macrophage. Source data are provided as a Source Data file.

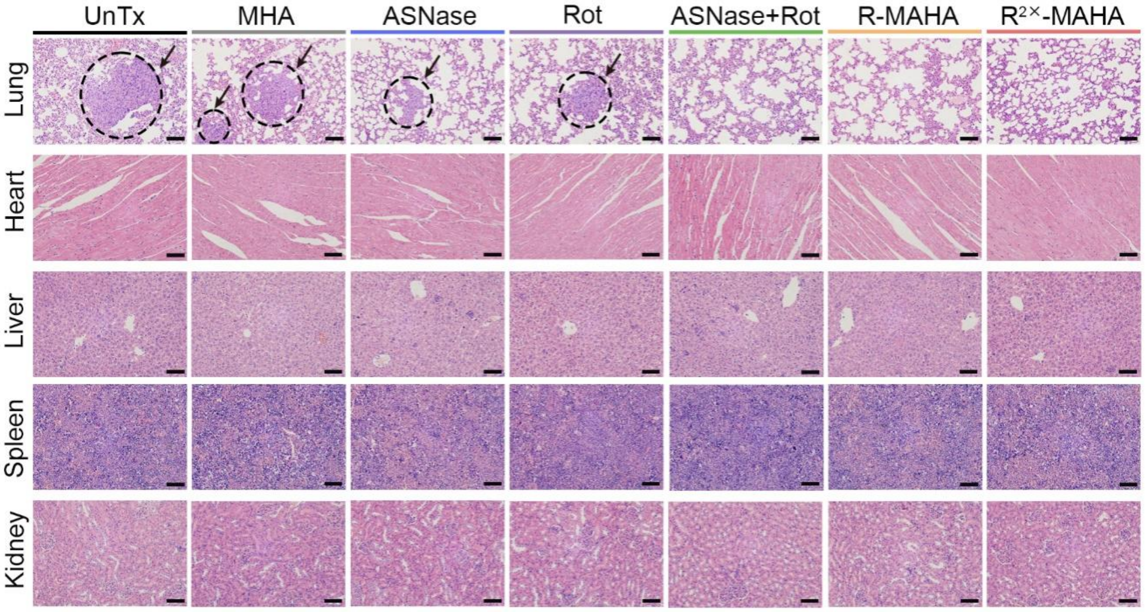

Supplementary Figure 26. Representative hematoxylin and eosin (H&E)-stained sections of major organs (lung, heart, liver, spleen, and kidney) from indicated groups. Metastatic tumors are indicated with black dotted circles. Scale bars: 100 μm. ASNase L-asparaginase, Rot rotenone.

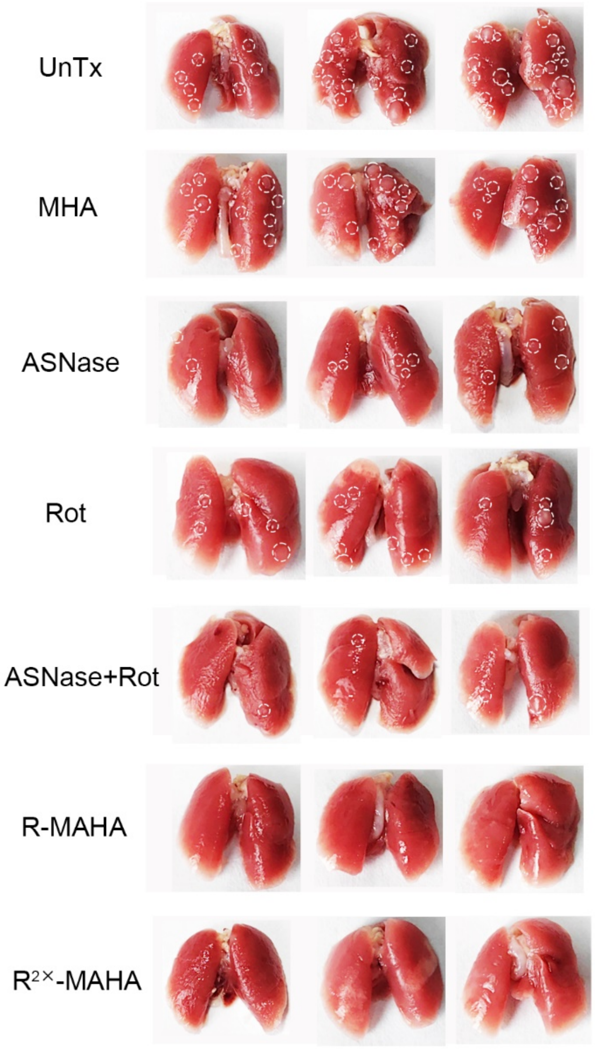

Supplementary Figure 27. Representative bright-field images of whole lungs from indicated groups. Metastatic tumors are indicated with white dotted circles. ASNase L-asparaginase, Rot rotenone.

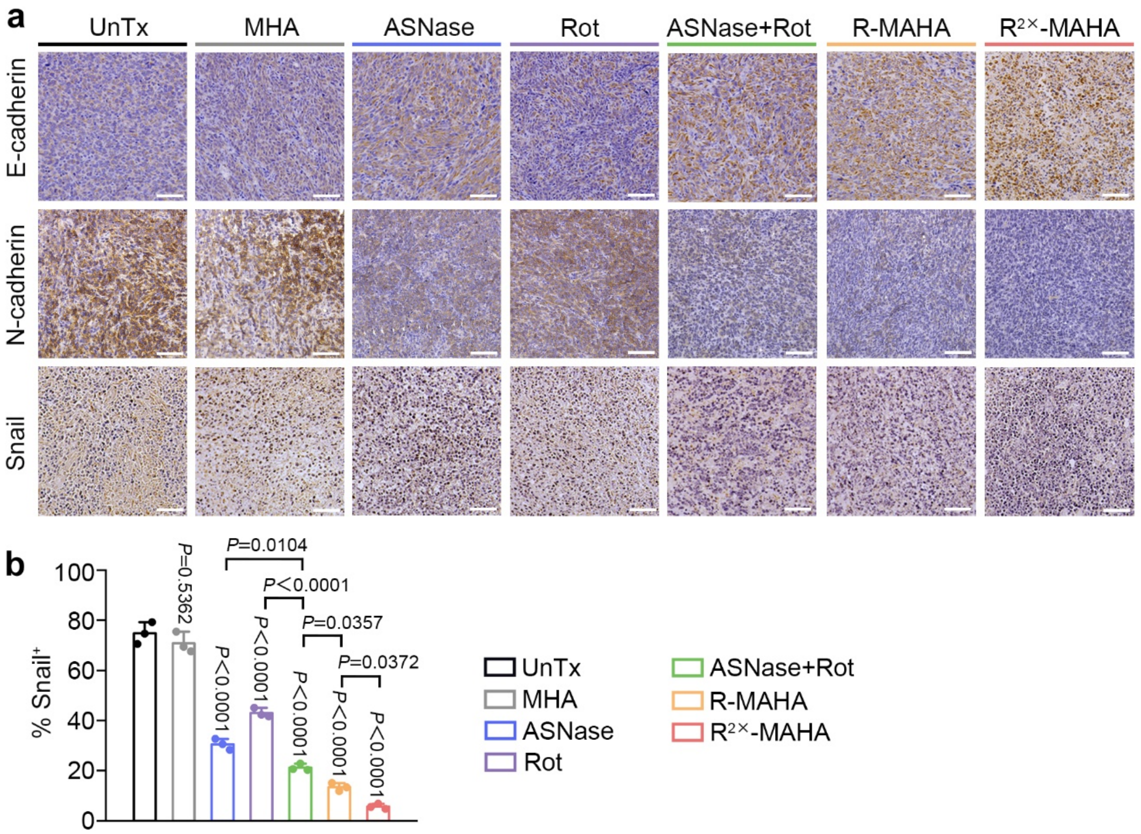

Supplementary Figure 28. Immunohistochemistry staining of EMT markers. (a) Representative images of immunohistochemistry staining for E-cadherin, N-cadherin or Snail in tumor sections after indicated treatments. Scale bars: 100 μm. (b) Expression levels of Snail in tumors after indicated treatments. Data are represented as mean ± SD (n = 3 independent samples). *P*-values were calculated using a one-way ANOVA followed by Tukey's post-hoc test. ASNase L-asparaginase, Rot rotenone. Source data are provided as a Source Data file.

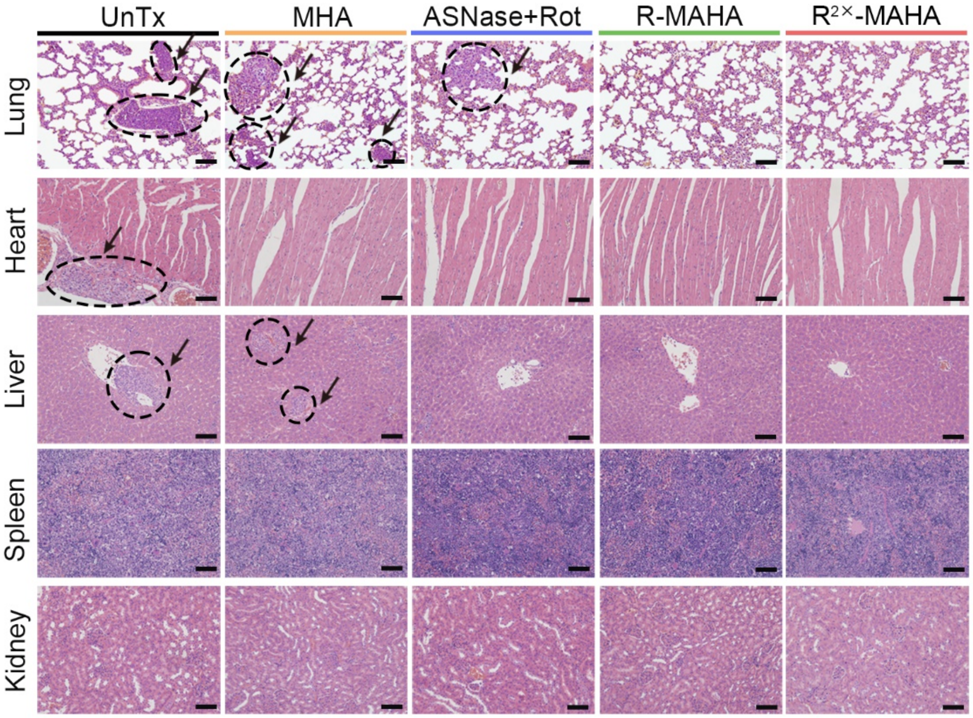

Supplementary Figure 29. Representative H&E-stained sections of major organs (lung, heart, liver, spleen, and kidney) from indicated groups. Metastatic tumors are indicated with black dotted circles. Scale bars: 100 μm. ASNase L-asparaginase, Rot rotenone.

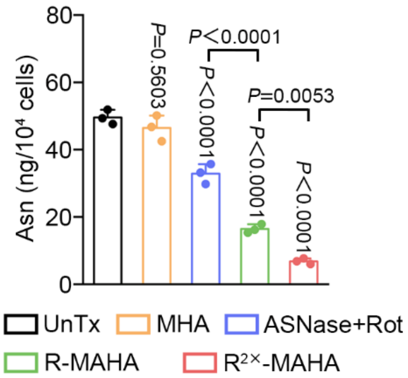

Supplementary Figure 30. Asn levels in tumor cells isolated from relapsed TNBC after indicated treatment. Data are represented as mean ± SD (n = 3 independent samples). *P*-values were calculated using a one-way ANOVA followed by Tukey's post-hoc test. Asn asparagine, ASNase L-asparaginase, Rot rotenone. Source data are provided as a Source Data file.

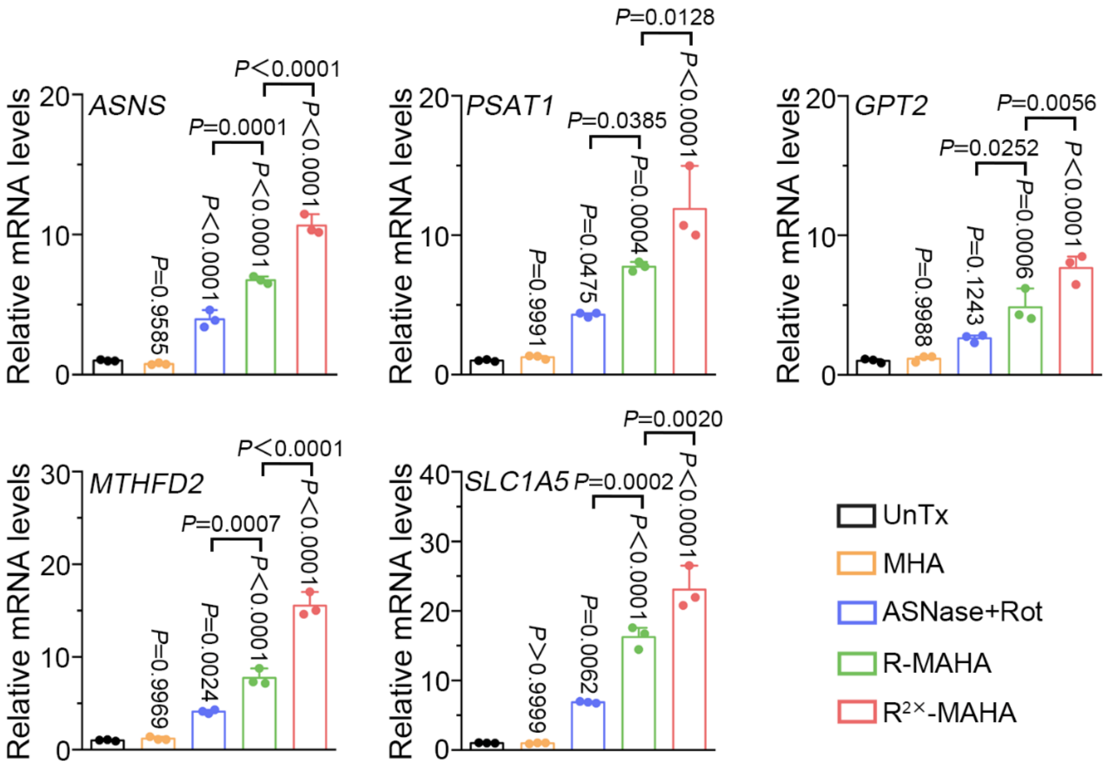

Supplementary Figure 31. Expression of ATF4-driven metabolic genes in tumor cells isolated from relapsed TNBC after indicated treatment. Data are represented as mean ± SD (n = 3 independent samples). *P*-values were calculated using a one-way ANOVA followed by Tukey's post-hoc test. ASNase L-asparaginase, Rot rotenone. Source data are provided as a Source Data file.

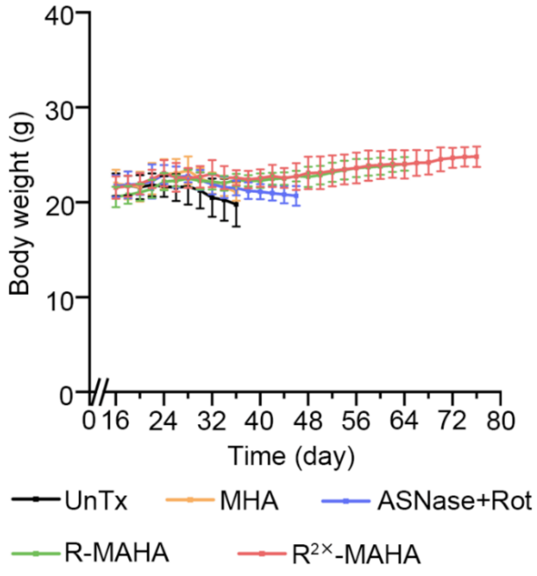

Supplementary Figure 32. Body weight of mice after indicated treatment. Data are represented as mean ± SD (n = 6 mice). ASNase L-asparaginase, Rot rotenone. Source data are provided as a Source Data file.

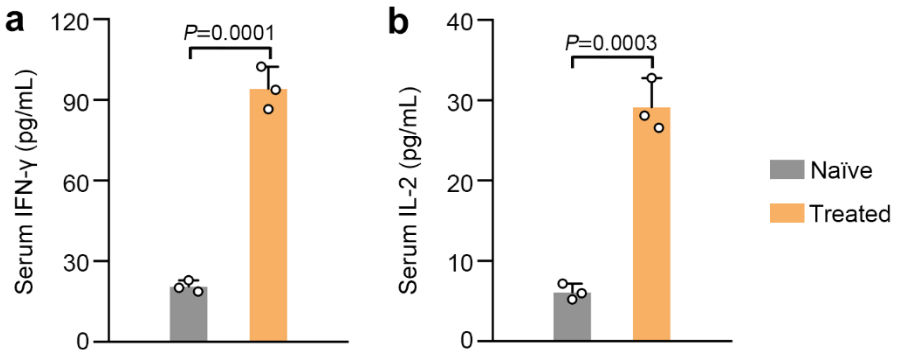

Supplementary Figure 33. Tcm cell activation. Serum levels of IFN-γ (a) and IL-2 (b) after 4T1 tumor rechallenge. Data are represented as mean ± SD (n = 3 independent samples). *P*-values were calculated using an unpaired two-tailed Student's *t*-test. IFN-γ interferon-γ, IL-2 interleukin-2. Source data are provided as a Source Data file.

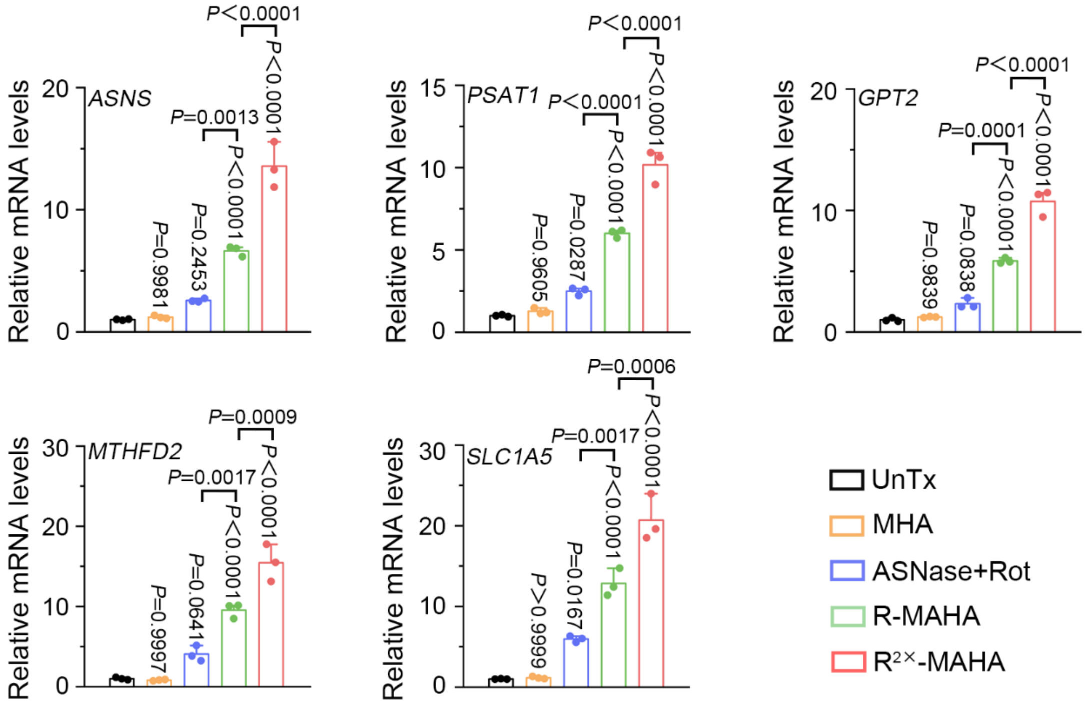

Supplementary Figure 34. Expression of ATF4-controlled metabolic genes in tumor cells isolated from relapsed CRC after indicated treatment. Data are represented as mean ± SD (n = 3 independent samples). *P*-values were calculated using a one-way ANOVA followed by Tukey’s post-hoc test. ASNase L-asparaginase, Rot rotenone. Source data are provided as a Source Data file.

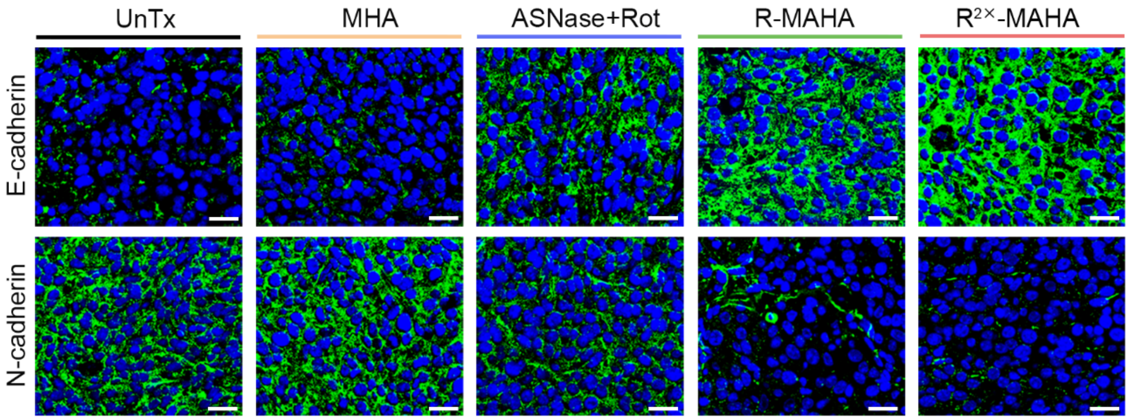

Supplementary Figure 35. Representative immunofluorescent images of E-cadherin or N-cadherin (green fluorescence) expression in relapsed CRC. DAPI (blue fluorescence) was used to label the nuclei. Scale bars: 20 μm. ASNase L-asparaginase, Rot rotenone.

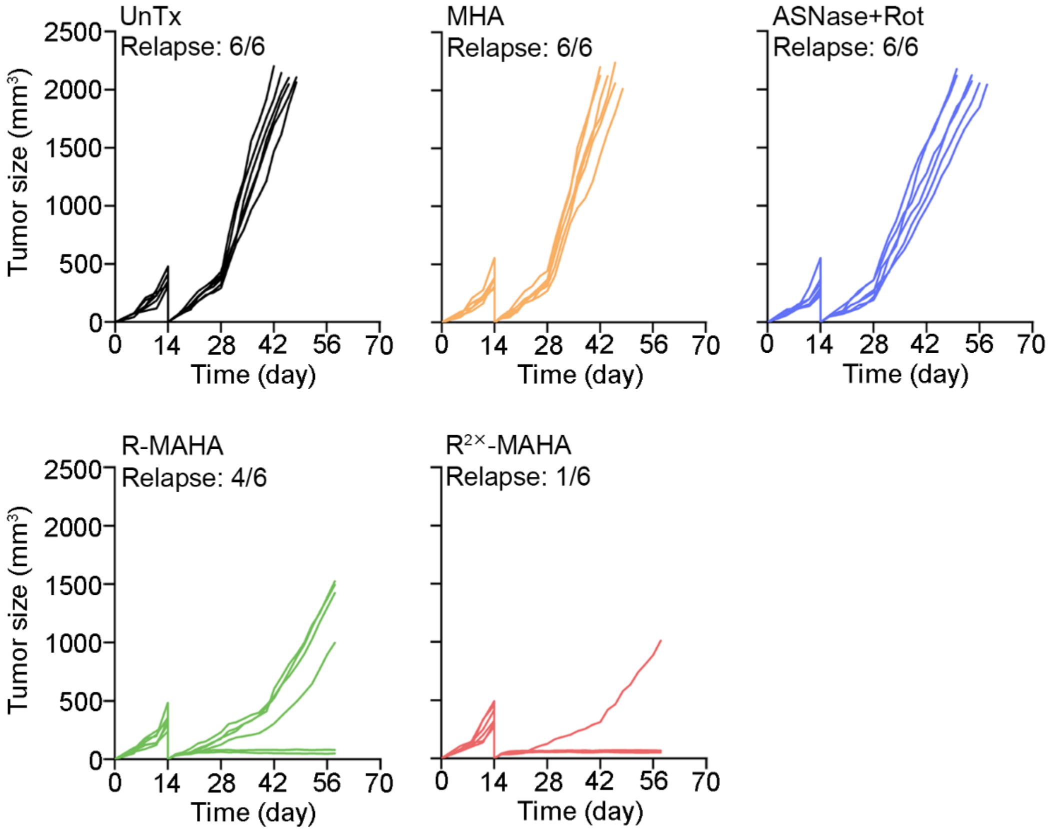

Supplementary Figure 36. Individual tumor growth kinetics of relapsed CRC after indicated treatment (n = 6 mice). ASNase L-asparaginase, Rot rotenone. Source data are provided as a Source Data file.

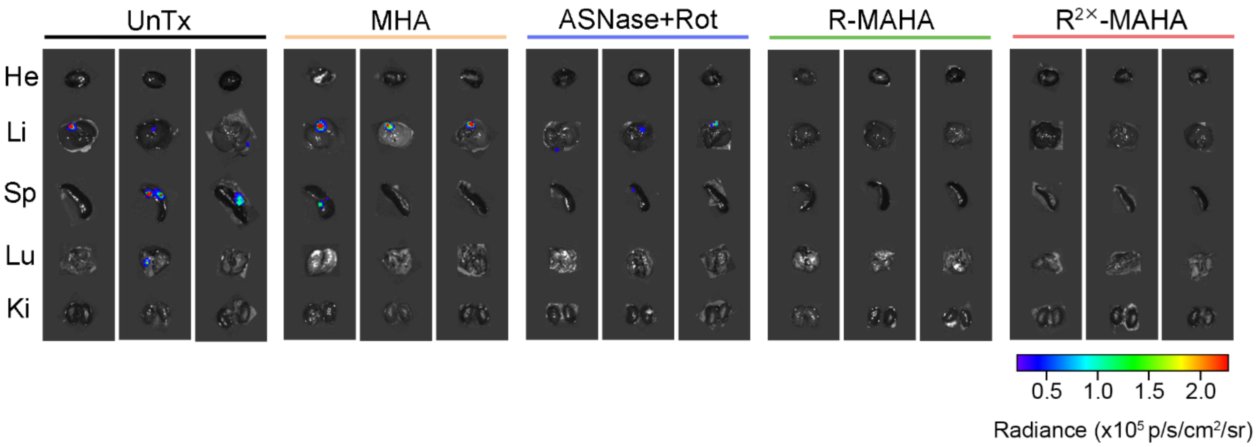

Supplementary Figure 37. Ex vivo bioluminescence images of major organs (n = 3 mice). ASNase L-asparaginase, Rot rotenone, He heart, Li liver, Sp spleen, Lu lung, Ki kidney.

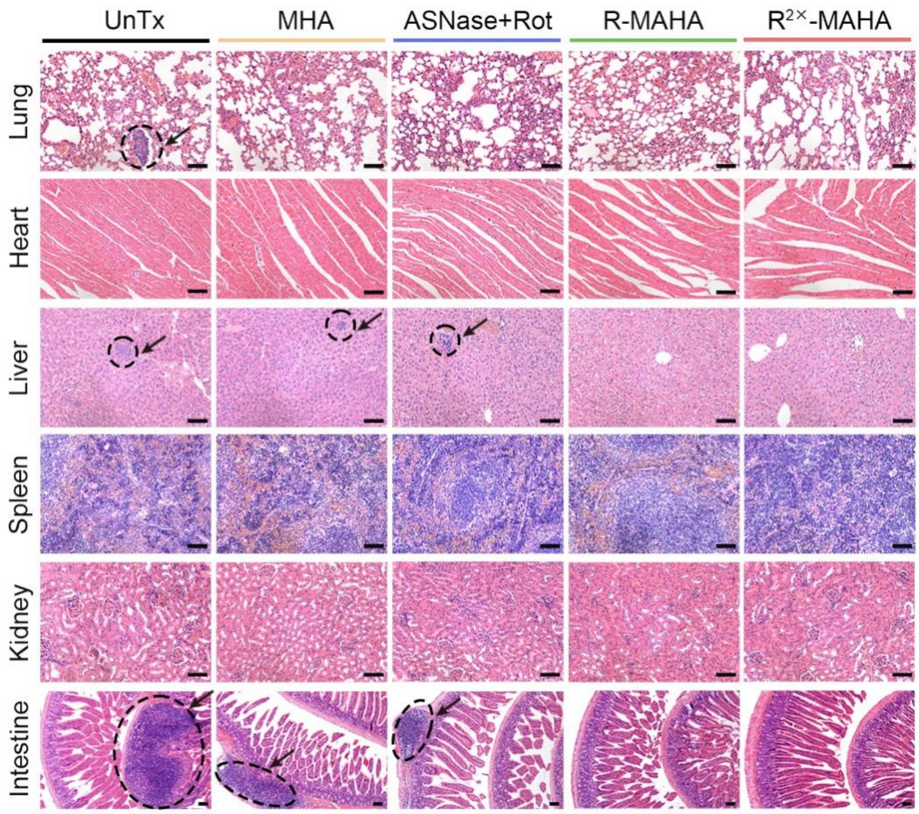

Supplementary Figure 38. Representative H&E-stained sections of major organs (lung, heart, liver, spleen, kidney, and intestine) from indicated groups. Metastatic tumors are indicated with black dotted circles. Scale bars: 100 μm.

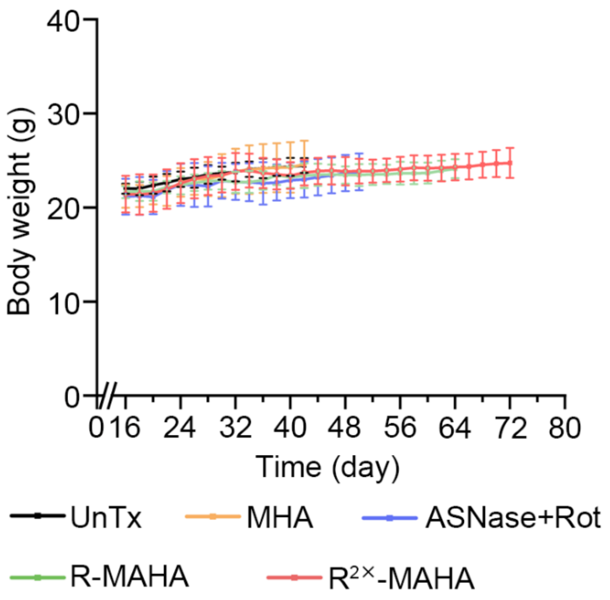

Supplementary Figure 39. Body weight of mice after indicated treatment. Data are represented as mean ± SD (n = 6 mice). ASNase L-asparaginase, Rot rotenone. Source data are provided as a Source Data file.

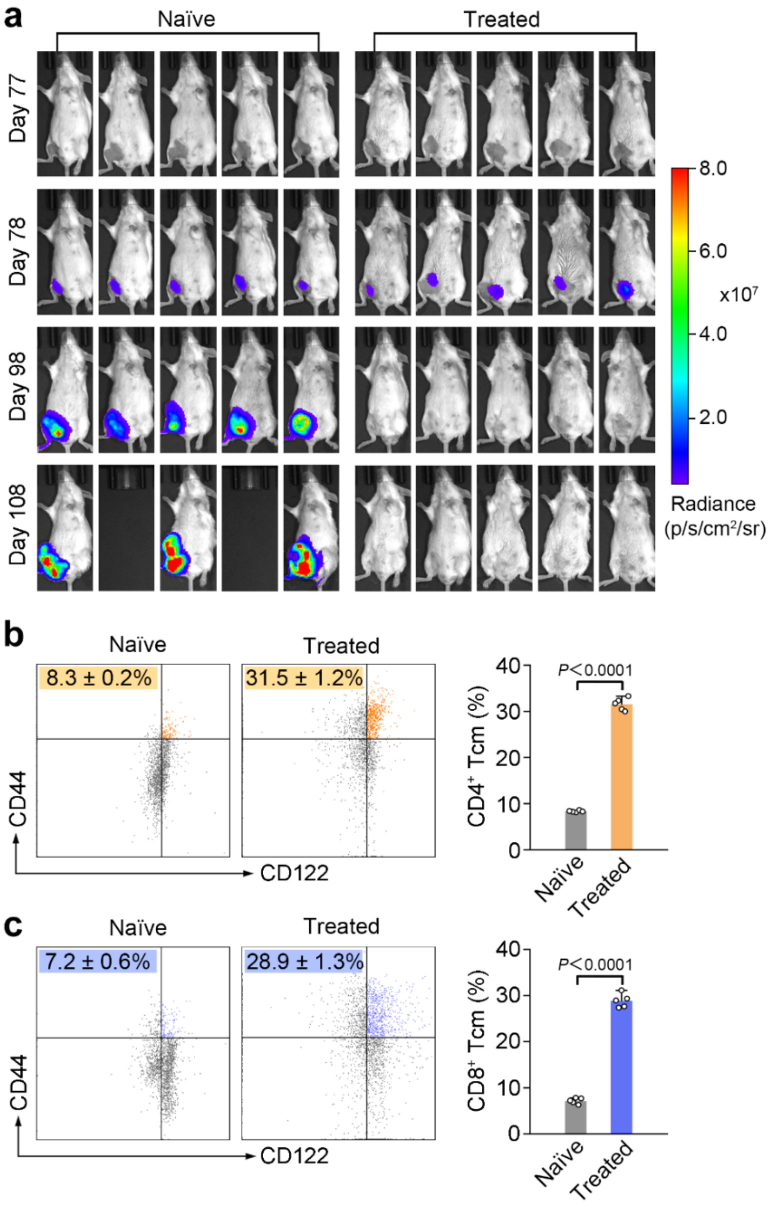

Supplementary Figure 40. Induction of T cell memory by R$^{2\times}$-MAHAs in post-surgical CRC model. (a) In vivo bioluminescence imaging of mice before (Day 77) and after (Day 78, Day 98, and Day 108) tumor rechallenge. (b, c) FACS analysis of CD4^{+} Tcm (gated on CD45^{+}CD4^{+} population) (b) and CD8^{+} Tcm cells (gated on CD45^{+}CD8^{+} population) (c) in splenocytes. Data are represented as mean ± SD (n = 5 independent samples). *P*-values were calculated using an unpaired two-tailed Student's *t*-test. Tcm central memory T. Source data are provided as a Source Data file.

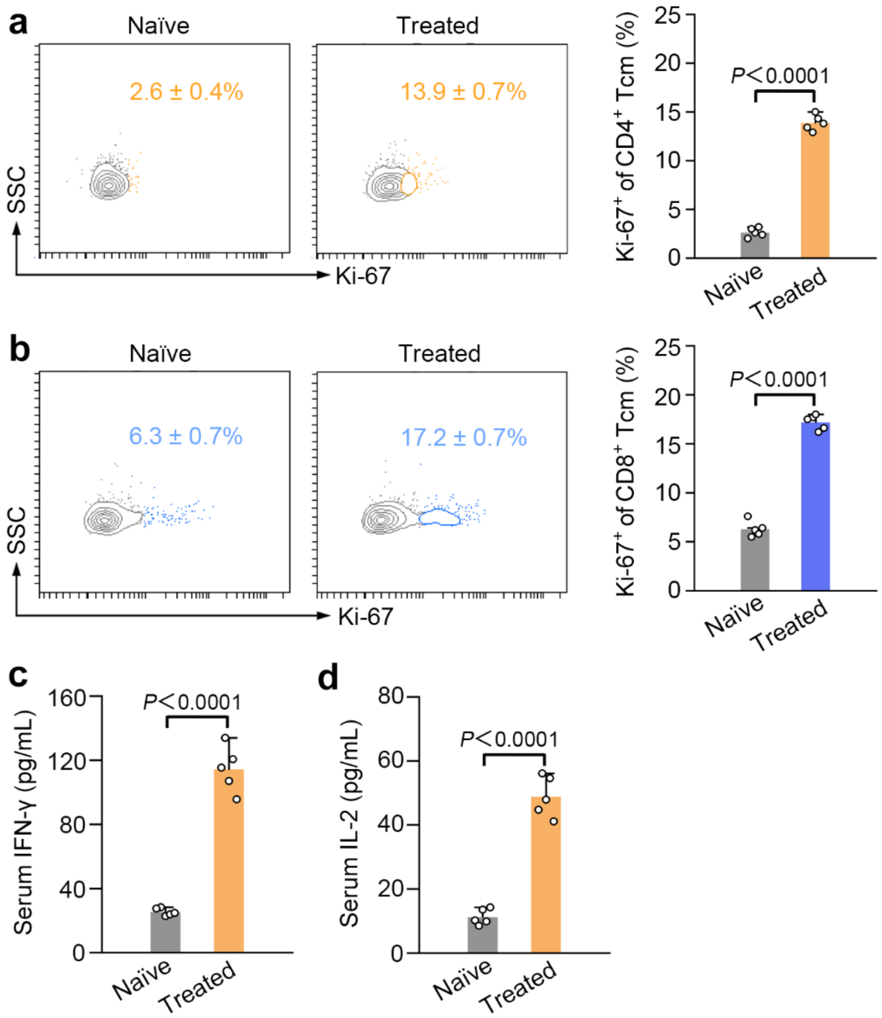

Supplementary Figure 41. Tcm cell proliferation and activation in CT26 rechallenged mice. (a, b) Intracellular Ki-67 expression in $CD4^+$ Tcm (gated on $CD45^+CD4^+CD44^+CD122^+$ population) (a) and $CD8^+$ Tcm cells (gated on $CD45^+CD8^+CD44^+CD122^+$ population) (b). (c, d) Serum levels of IFN-γ (c) and IL-2 (d) after tumor rechallenge. Data are represented as mean ± SD (n = 5 independent samples). *P*-values were calculated using an unpaired two-tailed Student's *t*-test. Tcm central memory T, IFN-γ interferon-γ, IL-2 interleukin-2. Source data are provided as a Source Data file.
