# Supplementary material for: Dual asparagine-depriving nanoparticles against solid tumors
Source: Nat Commun. 2025 Jul 1;16:5675. doi: 10.1038/s41467-025-60798-y (PMC12215482; doi:10.1038/s41467-025-60798-y)
Supplement: Supplementary file 2 — Reporting Summary [file 41467_2025_60798_MOESM2_ESM.pdf]

nature portfolio

Corresponding author(s): Prof. Haiyun Song

Last updated by author(s): May 23, 2025

Reporting Summary

Nature Portfolio wishes to improve the reproducibility of the work that we publish. This form provides structure for consistency and transparency in reporting. For further information on Nature Portfolio policies, see our Editorial Policies and the Editorial Policy Checklist.

Statistics

For all statistical analyses, confirm that the following items are present in the figure legend, table legend, main text, or Methods section.

n/a	Confirmed	
☐	☒	The exact sample size (*n*) for each experimental group/condition, given as a discrete number and unit of measurement
☐	☒	A statement on whether measurements were taken from distinct samples or whether the same sample was measured repeatedly
☐	☒	The statistical test(s) used AND whether they are one- or two-sided<br>*Only common tests should be described solely by name; describe more complex techniques in the Methods section.*
☒	☐	A description of all covariates tested
☐	☒	A description of any assumptions or corrections, such as tests of normality and adjustment for multiple comparisons
☐	☒	A full description of the statistical parameters including central tendency (e.g. means) or other basic estimates (e.g. regression coefficient) AND variation (e.g. standard deviation) or associated estimates of uncertainty (e.g. confidence intervals)
☐	☒	For null hypothesis testing, the test statistic (e.g. F, t, r) with confidence intervals, effect sizes, degrees of freedom and P value noted<br>*Give P values as exact values whenever suitable.*
☒	☐	For Bayesian analysis, information on the choice of priors and Markov chain Monte Carlo settings
☒	☐	For hierarchical and complex designs, identification of the appropriate level for tests and full reporting of outcomes
☐	☒	Estimates of effect sizes (e.g. Cohen's *d*, Pearson's *r*), indicating how they were calculated

Our web collection on statistics for biologists contains articles on many of the points above.

Software and code

Policy information about availability of computer code

Data collection	UV-Vis-NIR absorbance spectra (UV-1800, SHIMADZU)<br>ZEN3690 Zetasizer (Malvern)<br>Transmission electron microscope (JEM-1200EX, JEOL, Ltd., Japan)<br>Confocal laser scanning microscopy (CLSM, Leica TCS SP8)<br>IVIS spectrum imaging system (PerkinElmer)<br>Homogenizer (SCIENTZ-II D)<br>Fluorescence-activated cell sorting (BD FACSAria III and Beckman Coulter CytoFlex LX)<br>High-Speed Amino Acid Analyzer (LA8080 AminoSAAYA, Hitachi)
Data analysis	OriginPro (v 2021), Image J (v 1.53), FlowJo (v 10.8), GraphPad Prism (v 8.0)

For manuscripts utilizing custom algorithms or software that are central to the research but not yet described in published literature, software must be made available to editors and reviewers. We strongly encourage code deposition in a community repository (e.g. GitHub). See the Nature Portfolio guidelines for submitting code & software for further information.

Data

Policy information about availability of data

All manuscripts must include a data availability statement. This statement should provide the following information, where applicable:

- Accession codes, unique identifiers, or web links for publicly available datasets
- A description of any restrictions on data availability
- For clinical datasets or third party data, please ensure that the statement adheres to our policy

The authors declare that all the data supporting the findings of this study are available within the article, supplementary information or source data file. Source data are provided with this paper.

Research involving human participants, their data, or biological material

Policy information about studies with human participants or human data. See also policy information about sex, gender (identity/presentation), and sexual orientation and race, ethnicity and racism.

Reporting on sex and gender	N/A
Reporting on race, ethnicity, or other socially relevant groupings	N/A
Population characteristics	N/A
Recruitment	N/A
Ethics oversight	N/A

Note that full information on the approval of the study protocol must also be provided in the manuscript.

Field-specific reporting

Please select the one below that is the best fit for your research. If you are not sure, read the appropriate sections before making your selection.

☒ Life sciences ☐ Behavioural & social sciences ☐ Ecological, evolutionary & environmental sciences

For a reference copy of the document with all sections, see nature.com/documents/nr-reporting-summary-flat.pdf

Life sciences study design

All studies must disclose on these points even when the disclosure is negative.

Sample size	Sample sizes were chosen based on prior published studies and commonly accepted practices in the field.
Data exclusions	No data were excluded from the analyses.
Replication	Most experiments were performed at least three times independently. Animal experiments with duration longer than one month were performed at least twice independently.
Randomization	All samples/mice were randomly allocated into experimental groups.
Blinding	Since no population-based experiments were involved in this study, binding is not applicable for this study.

Reporting for specific materials, systems and methods

We require information from authors about some types of materials, experimental systems and methods used in many studies. Here, indicate whether each material, system or method listed is relevant to your study. If you are not sure if a list item applies to your research, read the appropriate section before selecting a response.

Materials & experimental systems

n/a	Involved in the study
☐	☒ Antibodies
☐	☒ Eukaryotic cell lines
☒	☐ Palaeontology and archaeology
☐	☒ Animals and other organisms
☒	☐ Clinical data
☒	☐ Dual use research of concern
☒	☐ Plants

Methods

n/a	Involved in the study
☒	☐ ChIP-seq
☐	☒ Flow cytometry
☒	☐ MRI-based neuroimaging

Antibodies

Antibodies used

anti-E-Cadherin Mouse mAb (Servicebio, Catalog: GB12083)
anti-N-Cadherin Mouse mAb (Servicebio, Catalog: GB12135)
anti-Snail Rabbit pAb (Servicebio, Catalog: GB11260)
anti-CD44 Rabbit pAb (Servicebio, Catalog: GB112054)
anti-CD45 Rabbit pAb (Servicebio, Catalog: GB113885)
Alexa Fluor™ 594 goat anti-rabbit secondary antibody (Invitrogen, Catalog: A11012)
anti-CD45 (BioLegend, Catalog: 103116, Clone: 30-F11)
anti-CD8 (BD Pharmingen, Catalog: 551162, Clone: 53-6.7)
anti-CD4 (BioLegend, Catalog: 100408, Clone: GK1.5)
anti-IFN-γ (BioLegend, Catalog: 505826, Clone: XMG1.2)
anti-CD11c (BioLegend, Catalog: 117308, Clone: N418)
anti-CD80 (BioLegend, Catalog: 104721, Clone: 16-10A1)
anti-CD86 (BioLegend, Catalog: 105031, Clone: GL-1)
anti-CD25 (BD Horizon, Catalog: 562606, Clone: PC61)
anti-Foxp3 (eBioscience, Catalog: 25577382, Clone: FJK-16s)
anti-CD11b (BioLegend, Catalog: 101228, Clone: M1/70)
anti-F4/80 (BioLegend, Catalog: 123110, Clone: BM8)
anti-CD206 (BioLegend, Catalog: 141717, Clone: C068C2)
anti-CD44 (BioLegend, Catalog: 103040, Clone: IM7)
anti-CD122 (BioLegend, Catalog: 123216, Clone: TM-β1)
anti-Ki-67 (BioLegend, Catalog: 652423, Clone: 16A8)
anti-Ki-67 (BioLegend, Catalog: 652403, Clone: 16A8)

Validation

Validation of each antibody was done under standard information offered by the supplier.
-Anti-E-Cadherin Mouse mAb has been validated by the company in IHC and IF (Servicebio, Catalog: GB12083, 1:500)
-Anti-N-Cadherin Mouse mAb has been validated by the company in western blots, IHC and IF (Servicebio, Catalog: GB12135, 1:500)
-Anti-Snail Rabbit pAb has been validated by the company in IHC and IF (Servicebio, Catalog: GB11260, 1:400)
-Anti-CD44 Rabbit pAb has been validated by the company in western blots, IHC and IF (Servicebio, Catalog: GB112054, 1:500)
-Anti-CD45 Rabbit pAb has been validated by the company in western blots, IHC and IF (Servicebio, Catalog: GB113885, 1:500)

Eukaryotic cell lines

Policy information about cell lines and Sex and Gender in Research

Cell line source(s)

4T1 breast cancer cells (TCM32), CT26 colorectal cancer cells (TCM37) and LLC lung cancer cells (TCM47) were purchased from Cell Bank of Chinese Academy of Sciences (Shanghai).
H22 hepatic cancer cells (CVCL_H613) and Panc02 pancreatic cancer cells (CVCL_D627) were purchased from Fuheng Biology Science and Technology Co., Ltd. (Shanghai).

Authentication

All cell lines were authenticated by short tandem repeat (STR) analysis.

Mycoplasma contamination

All cell lines were tested for mycoplasma routinely.

Commonly misidentified lines
(See ICLAC register)

No commonly misidentified cell line was used.

Animals and other research organisms

Policy information about studies involving animals; ARRIVE guidelines recommended for reporting animal research, and Sex and Gender in Research

Laboratory animals

Female BALB/c mice (6-8 weeks) were purchased from SLAC Laboratory Animal Co. Ltd (Shanghai, China). The mice were kept in a barrier environment with a constant temperature of 24°C and a relative humidity of 50%. The mice were maintained under a 12-hour light and 12-hour dark cycle.

Wild animals	No wild animals were used in this study.
Reporting on sex	Although we only selected female mice for this study, the issue of sex was not within our considerations. We chose a single sex to ensure consistency in the experimental process, which is more beneficial for comparing between groups.
Field-collected samples	No field-collected samples were used in this study.
Ethics oversight	All mouse experiments were reviewed and approved by the Animal Care and Use Committee of Shanghai Jiao Tong University School of Medicine (A-2022-115). A maximum tumor size of 20 mm in any dimension, as approved by the ethics committee, was not exceeded during the study. The mice were euthanized when their tumors reached a volume of approximately 2000 mm3, with any dimension below 20 mm.

Note that full information on the approval of the study protocol must also be provided in the manuscript.

Plants

Seed stocks	N/A
Novel plant genotypes	N/A
Authentication	N/A

Flow Cytometry

Plots

Confirm that:

☒ The axis labels state the marker and fluorochrome used (e.g. CD4-FITC).

☒ The axis scales are clearly visible. Include numbers along axes only for bottom left plot of group (a 'group' is an analysis of identical markers).

☒ All plots are contour plots with outliers or pseudocolor plots.

☒ A numerical value for number of cells or percentage (with statistics) is provided.

Methodology

Sample preparation	See section "Immune cell analysis" in Methods.
Instrument	Fluorescence-activated cell sorting (BD FACSAria III or Beckman Coulter CytoFlex LX)
Software	FlowJo 10.8.0
Cell population abundance	The CD8+ T cells, IFN-γ+CD8+ T cells, CD80+CD86+ DCs, Treg cells, and M2-TAMs within tumors were gated as CD45+CD8+, CD45+CD8+IFN-γ+, CD45+CD11c+CD80+CD86+, CD45+CD4+CD25+Foxp3+, and CD45+CD11b+F4/80+CD206+ cells, respectively.<br>CD4+ Tcm, CD8+ Tcm, Ki-67+CD4+ Tcm, and Ki-67+CD8+ Tcm populations were characterized as the CD45+CD4+CD44+CD122+, CD45+CD8+CD44+CD122+, CD45+CD4+CD44+CD122+Ki-67+, and CD45+CD8+CD44+CD122+Ki-67+ cells according to the previously reported work.
Gating strategy	See section "Immune cell analysis" in Methods.

☐ Tick this box to confirm that a figure exemplifying the gating strategy is provided in the Supplementary Information.
